# The c‐Src/LIST Positive Feedback Loop Sustains Tumor Progression and Chemoresistance

**DOI:** 10.1002/advs.202300115

**Published:** 2023-05-08

**Authors:** Xianteng Wang, Bing Wang, Fang Li, Xingkai Li, Ting Guo, Yushun Gao, Dawei Wang, Weiren Huang

**Affiliations:** ^1^ Department of Urology Shenzhen Institute of Translational Medicine Shenzhen Second People's Hospital The First Affiliated Hospital of Shenzhen University Guangdong Key Laboratory for Biomedical Measurements and Ultrasound Imaging National‐Regional Key Technology Engineering Laboratory for Medical Ultrasound School of Biomedical Engineering Shenzhen University Medical school Shenzhen 518060 China; ^2^ Shenzhen Institute of Synthetic Biology Shenzhen Institutes of Advanced Technology Chinese Academy of Sciences Shenzhen 518055 China; ^3^ Guangdong Key Laboratory of Systems Biology and Synthetic Biology for Urogenital Tumors Shenzhen Second People's Hospital The First Affiliated Hospital of Shenzhen University Shenzhen 518035 China; ^4^ Department of Thoracic Surgery National Cancer Center/National Clinical Research Center for Cancer/Cancer Hospital Chinese Academy of Medical Sciences and Peking Union Medical College Beijing 100021 China; ^5^ Department of Thoracic Surgery National Cancer Center/National Clinical Research Center for Cancer/Hebei Cancer Hospital Chinese Academy of Medical Sciences Langfang 065001 China; ^6^ Department of Thoracic Surgery Chifeng Municipal Hospital Chifeng 024000 China

**Keywords:** c‐Src, cancer, chemoresistance, epigenetic, *LIST*, P65

## Abstract

Chemotherapy resistance and treatment failure hinder clinical cancer treatment. *Src*, the first mammalian proto‐oncogene to be discovered, is a valuable anti‐cancer therapeutic target. Although several c‐Src inhibitors have reached the clinical stage, drug resistance remains a challenge during treatment. Herein, a positive feedback loop between a previously uncharacterized long non‐coding RNA (lncRNA), which the authors renamed lncRNA‐inducing c‐Src tumor‐promoting function (*LIST*), and c‐Src is uncovered. *LIST* directly binds to and regulates the Y530 phosphorylation activity of c‐Src. As a c‐Src agonist, *LIST* promotes tumor chemoresistance and progression in vitro and in vivo in multiple cancer types. c‐Src can positively regulate *LIST* transcription by activating the NF‐*κ*B signaling pathway and then recruiting the P65 transcription factor to the *LIST* promoter. Interestingly, the *LIST*/c‐Src interaction is associated with evolutionary new variations of c‐Src. It is proposed that the human‐specific *LIST*/c‐Src axis renders an extra layer of control over c‐Src activity. Additionally, the *LIST*/c‐Src axis is of high physiological relevance in cancer and may be a valuable prognostic biomarker and potential therapeutic target.

## Graphical abstract

This study provides novel insights into the exploration of efficient c‐Src agonists at the level of epigenetic regulation. lncRNA *LIST* and c‐Src are involved in a positive feedback regulatory loop during tumor resistance. Evolutionarily, this human‐specific c‐Src/LIST axis renders an additional yet critical layer of control over the c‐Src activity.

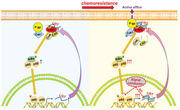

## 1 Introduction

Chemoresistance is considered the main cause of cancer therapy failure, leading to relapse and metastasis.^[^ ^1^, ^2^ ^]^ Therefore, chemoresistance‐associated molecular pathways must be elucidated and novel therapeutic approaches must be identified for cancer therapy.

c‐Src is a non‐receptor tyrosine kinase in which Tyr530 and Tyr419 are the most important phosphorylation sites.^[^ ^3^ ^]^ In the resting state, c‐Src is phosphorylated at Tyr530, where it is present in an inactive form. When stimulated by an external signal, c‐Src dephosphorylates at Tyr530, resulting in a conformational change to activate Tyr419 autophosphorylation and subsequent activation of kinase activity.^[^ ^4^, ^5^ ^]^ Thus, dephosphorylation of Y530 is a critical step for c‐Src activation and signal transmission. c‐Src is significantly activated (Tyr530 dephosphorylation) in various tumors.^[^ ^6^ ^]^ Inhibition of c‐Src phosphorylation at Tyr530 can promote tumor progression and chemoresistance.^[^ ^7^ ^]^ Several kinase inhibitors targeting c‐Src phosphorylation sites have been used in clinical trials.^[^ ^8^, ^9^ ^]^ However, tumor cells often acquire drug resistance during treatment. Therefore, it is necessary to investigate the role of c‐Src in tumor‐acquired drug resistance and develop novel strategies for cancer therapy.

Numerous studies over the past few years have demonstrated that long noncoding RNAs (lncRNAs) can function as oncogenes^[^ ^10^, ^11^, ^12^, ^13^ ^]^ or tumor suppressors^[^ ^14^, ^15^, ^16^ ^]^ to participate in tumor progression and chemoresistance through a variety of mechanisms, such as epigenetic, transcriptional, and post‐transcriptional regulation.^[^ ^17^, ^18^, ^19^, ^20^, ^21^, ^22^ ^]^ However, the lncRNAs that directly regulate c‐Src activity during cancer development remain largely unknown.

In this study, we explored potential lncRNAs that might influence c‐Src activity and identified a lncRNA (gene name: RP11‐713M15.2, transcript ID: ENST00000605955.1) that directly binds to and inhibits c‐Src Y530 phosphorylation. Compared with normal tissues, lncRNA RP11‐713M15.2 was relatively highly expressed in multiple types of tumors, especially in drug‐resistant tumor tissues. We demonstrated that the activity of c‐Src depends on lncRNA RP11‐713M15.2. In fact, by inhibiting c‐Src Y530 phosphorylation directly, RP11‐713M15.2 strongly promoted tumor progression and chemoresistance. Therefore, we propose changing the name of the lncRNA RP11‐713M15.2 to lncRNA‐inducing c‐Src tumor‐promoting function (*LIST*). Further investigation revealed that c‐Src positively regulates the transcription of *LIST* by activating the NF‐*κ*B signaling pathway.

In summary, we uncovered a positive feedback loop between the critical proto‐oncogene c‐Src and a previously uncharacterized lncRNA *LIST*, and we identified a novel and efficient c‐Src agonist from the perspective of epigenetic regulation.

## 2 Results

### 2.1 Screening and Identification of the lncRNAs Binding to c‐Src

**Figure 1:**
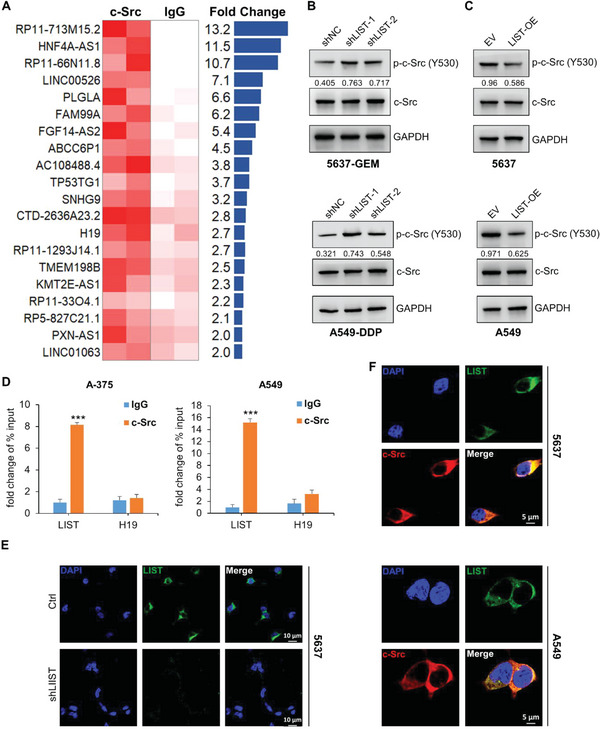
Identification of lncRNAs that bind and regulate c‐Src phosphorylation. A) Expression profiles of lncRNAs pulled down by c‐Src RIP in cell line 5637; IgG‐RIP was used as a negative control. The lncRNAs (fold‐change ≥ 2) are included in the table. Experiments were performed using two biological replicates. B,C) The total protein and phosphorylation levels of c‐Src were detected by western blotting upon *LIST* knockdown or overexpression. The proteins were quantified by Image J software. The numbers represent the ratio of p‐c‐Src‐Y530/c‐Src. D) *LIST* expression was measured via c‐Src RIP‐qPCR in A‐375 and A‐549 cancer cells. H19 was used as the negative control. The error bars represent the standard deviation of three replicates (***p < 0.001), Student's *t*‐test. E) The subcellular localization of *LIST* (green) was examined via RNA FISH assay in cell line 5637 under control and *LIST* knockdown conditions. DAPI (blue) represents nuclei. Scale bar: 10µm. F) Colocalization immunofluorescence staining of *LIST* (green) and c‐Src (red) in 5637 and A‐549 cancer cells. Nuclei were stained with DAPI (blue). Scale bar: 5µm.

To identify lncRNAs that are directly involved in c‐Src activity in cancer, we first screened c‐Src‐bound lncRNAs in bladder cancer cells (cell line 5637) using an RNA‐binding protein immunoprecipitation (RIP) assay combined with lncRNA sequencing. LncRNAs with a 3‐fold change were selected for further verification (**Figure** **1**A). Next, we screened lncRNAs that affect the sensitivity of cell line 5637 to c‐Src inhibition by monitoring cell viability. The results showed that knockdown of the five lncRNAs (RP11‐713M15.2, PLGLA, ABCC6P1, AC108488.4, and SNHG9) increased the sensitivity of cells to c‐Src inhibition (Figure S1A–C, Supporting Information). Dephosphorylation of Y530 is necessary for c‐Src activity. Interestingly, we found that only repression of RP11‐713M15.2, the most enriched binding lncRNA of c‐Src, dramatically increased the phosphorylation level at the Y530 site of c‐Src (Figure S1D, Supporting Information). Moreover, the re‐expression of RP11‐713M15.2 restored the decreased cell viability caused by RP11‐713M15.2 knockdown in the presence of a c‐Src inhibitor (Figure S1E, Supporting Information). This suggests that RP11‐713M15.2 (renamed *LIST*) is the lncRNA that most likely regulates c‐Src activity.

We first accurately determined that the transcript length of *LIST* is 1129 nt through 5ʹ and 3ʹ Rapid‐amplification of cDNA ends (RACE) assays, and that the genome location is at chr8: 120812219‐120813347 (Figure S2, Supporting Information). The Cancer Genome Atlas (TCGA) data analysis showed that *LIST* expression increased in a variety of tumors compared with adjacent normal tissues, especially in lung cancer, bladder urothelial carcinoma, prostate adenocarcinoma, and melanoma (Figure S3, Supporting Information). In the corresponding tumor cell lines, 5637 (bladder cancer), A549 (lung cancer), and A375 (melanoma), the RNA copy number of *LIST* was ≈100 per cell (Figure S4, Supporting Information). Interestingly, the *LIST* RNA copy number increased by ≈3‐fold when these cells developed drug resistance (Figure S4, Supporting Information). Therefore, we performed a knockdown of *LIST* via shRNA in chemoresistant cell lines (Figure S5A, Supporting Information), and stably overexpressed lentivirus *LIST* in chemosensitive cells (Figure S5B, Supporting Information). The results showed that *LIST* knockdown dramatically enhanced the phosphorylation level at the Y530 site of c‐Src (Figure 1B and Figure S6A, Supporting Information), while overexpression of *LIST* reduced the phosphorylation of c‐Src (Tyr530) (Figure 1C and Figure S6B, Supporting Information).

Next, a RNA binding protein immunoprecipitation (RIP) assay using the c‐Src antibody followed by qRT‐PCR confirmed the binding of c‐Src and *LIST* in both lung cancer and melanoma cells (Figure 1D). Growing evidence suggests that lncRNA function is related to the unique subcellular localization patterns of the lncRNA.^[^ ^23^ ^]^ We found that endogenous *LIST* was mainly localized in the cytoplasm using fluorescence in situ hybridization (FISH) and subcellular fractionation, followed by qRT‐PCR (Figure 1E and Figure S7, Supporting Information). Notably, the knockdown of *LIST* resulted in a significant reduction in the cytoplasm, as confirmed by the FISH assay (Figure 1E). Immunofluorescence imaging further revealed the co‐localization of c‐Src and *LIST* in the cytoplasm (Figure 1F and Figure S6C,D, Supporting information). These results indicate that *LIST* directly binds to c‐Src and inhibits its Y530 phosphorylation level.

### 2.2 Characterization of the c‐Src‐*LIST* Interaction

**Figure 2:**
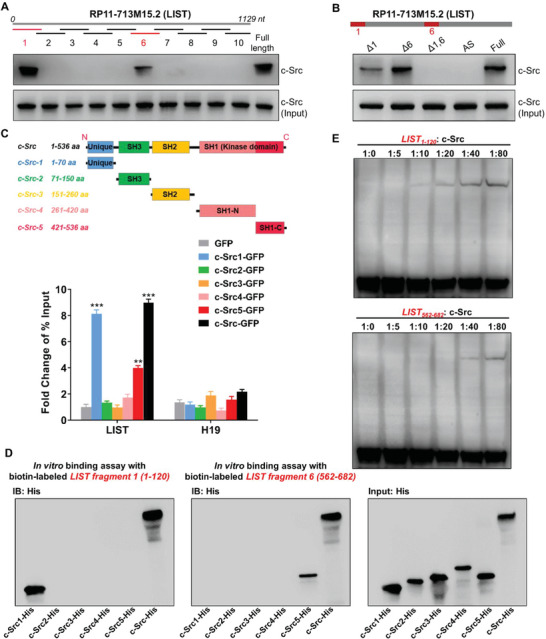
Direct interaction between *LIST* and c‐Src. A) Interactions between fragmented *LIST* and c‐Src. Biotin‐labeled *LIST* fragments (≈120 nt each) and full‐length *LIST* were used for protein pull‐down in cell line 5637, followed by western blot analysis of c‐Src. B) Interactions between *LIST* truncation and c‐Src. biotin‐labeled *LIST* truncations (that lack one or both of fragments 1 and 6) and full‐length *LIST* were used for protein pull‐down in cell line 5637, followed by western blot analysis of c‐Src. C) Different c‐Src truncations were fused with GFP and expressed in cell line 5637. Enrichment of *LIST* was detected using GFP‐RIP‐qPCR. H19 was used as a negative control. The error bars represent the SD of three replicates. D) The biotin‐labeled *LIST* fragment (1 or 6) was incubated with Dynabeads (MyOne Streptavidin C1), and then mix with the purified His‐tagged c‐Src truncations and full‐length proteins in binding buffer, followed by western blotting for His. E) EMSA image of a biotin‐labeled *LIST* fragment (1 or 6) binding to different concentration gradients of c‐Src proteins. Two fmol of the labeled *LIST* fragment were used for each EMSA reaction.

To further dissect the binding mechanism between *LIST* and c‐Src, we constructed a series of biotin‐labeled *LIST* fragments for protein pull‐down followed by immunoblotting for c‐Src. The results revealed that fragment 1 (1‐120 nt) and fragment 6 (562‐682 nt) of *LIST* are responsible for c‐Src binding (**Figure** **2**A). *LIST* fragment 1 exhibited a binding capability similar to that of full‐length *LIST*, while fragment 6 showed a significantly weaker binding ability. Additionally, removing fragment 1 remarkably attenuated c‐Src binding, but deleting fragment 6 had little effect on c‐Src binding, indicating that fragment 1 may be dominant in binding to c‐Src (Figure 2B).

The c‐Src protein consists of a unique domain, SH3 domain, SH2 domain, and kinase activity domain (SH1) from the N‐terminal to the C‐terminal.^[^ ^8^, ^24^ ^]^ The RIP assay using numerous exogenously expressed shortened isoforms of c‐Src confirmed that the unique domain and the SH1‐C domain were responsible for binding to c‐Src, of which the unique domain was significantly stronger than the SH1‐C domain (Figure 2C). To further clarify the binding region of *LIST* to c‐Src, we performed in vitro binding assays using biotin‐labeled *LIST* fragments (1 and 6) with purified His‐tagged c‐Src truncated proteins. As shown in Figure 2D, *LIST* fragment 1 was bound to the unique domain of c‐Src while *LIST* fragment 6 was responsible for binding with the SH1‐C domain, indicating that *LIST* may fold into a higher‐order structure for binding to the c‐Src multi‐domain. In addition, we conducted an electrophoretic mobility shift assay (EMSA) between biotin‐labeled *LIST* fragments (1 and 6) and c‐Src. As expected, both *LIST* fragments 1 and 6 could bind c‐Src, but fragment 1 had a higher affinity for c‐Src than fragment 6 (Figure 2E).

Both in vitro and in vivo assays confirmed that *LIST* is capable of binding directly to c‐Src. As a result, we decided to conduct additional research to determine whether the molecular and cellular functions of c‐Src are associated with *LIST*.

### 2.3 Expression and Functional Implication of *LIST* in Human Cancers

**Figure 3:**
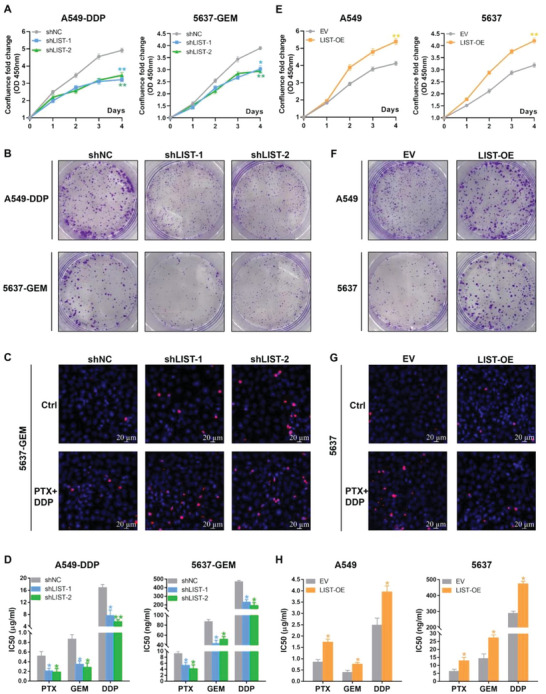
The function of *LIST* in cancer cell proliferation, apoptosis, and chemoresistance. A) CCK‐8 assay was used to analyze the proliferation of chemoresistant cancer cells with *LIST* knockdown. The error bars represent the SD of three replicates (*p < 0.05, **p < 0.01), Student's *t*‐test. B) Colony formation assay was performed to detect the proliferation capacity of chemoresistant cancer cells with *LIST* knockdown. Refer to Figure S9A, Supporting Information for the statistical analyses. C) TUNEL assay was performed to detect cell apoptosis (red) upon *LIST* knockdown. Refer to Figure S9B, Supporting Information for the statistical analyses. PTX (paclitaxel), 0.05 µM; DDP (cisplatin), 5µM. D) The drug IC_50_ value was examined using the CellTiter‐Glo Kit upon *LIST* knockdown. The error bars represent the SD of three replicates (*p < 0.05, **p < 0.01), Student's *t*‐test. E) Proliferation of chemosensitive cancer cells stably transfected with *LIST* overexpression via the CCK‐8 assay. The error bars represent the SD of three replicates (**p < 0.01), Student's *t*‐test. F) Cell proliferation capacity of *LIST*‐overexpressing cells was detected using a colony formation assay. Refer to Figure S9D, Supporting Information for the statistical analyses. G) Apoptotic cells (red) were stained using a TUNEL assay upon *LIST* overexpression. Refer to Figure S9E, Supporting Information for the statistical analyses. PTX (paclitaxel), 0.05µM; DDP (cisplatin), 5µM. H) The drug IC_50_ value of cancer cells stably transfected with *LIST* overexpression was examined using the CellTiter‐Glo Kit. The error bars represent the SD of three replicates (*p < 0.05), Student's *t*‐test.

Since *LIST* can regulate the phosphorylation activity of c‐Src, we investigated whether *LIST* has any discernible impact on tumor progression and chemoresistance. We first performed a knockdown of *LIST* via shRNA in chemoresistant cell lines (Figure S5A, Supporting Information), and a significant decrease in the proliferative capacity of these cells was observed (**Figure** **3**A and Figure S8A, Supporting Information). *LIST* knockdown also resulted in reduced colony formation in these cells (Figure 3B, and Figures S8B and S9A, Supporting Information). Additionally, TdT‐mediated dUTP nick end labeling (TUNEL) assays showed that *LIST* downregulation increased the apoptotic rate of these cells, especially when treated with chemotherapeutic drugs (Figure 3C, and Figures S8C and S9B,C, Supporting Information). Furthermore, the IC_50_ values were analyzed by measuring the sensitivity of these cells to various chemotherapeutic drugs and revealed an ≈2‐fold decrease in *LIST*‐knockdown cells (Figure 3D and Figure S8D, Supporting Information). In contrast, chemosensitive cells with stable lentivirus *LIST* overexpression (Figure S5B, Supporting Information) exhibited notable increases in cell proliferation (Figure 3E and Figure S8E, Supporting Information) and colony formation (Figure 3F, and Figures S8F and S9D, Supporting Information). Consistently, the proportion of apoptotic cells conspicuously decreased in the *LIST* overexpression group when treated with chemotherapeutic drugs (Figure 3G, and Figures S8G and S9E,F, Supporting Information). Cells overexpressing *LIST* also showed an ≈2‐fold improvement in IC_50_ values (Figure 3H and Figure S8H, Supporting Information).

We further discovered that overexpression of a deletion construct of *LIST* devoid of the two c‐Src‐binding fragments (1 and 6) had no effect on cell proliferation and drug resistance when compared to overexpression of full‐length *LIST*, and simply expressing the two fragments together also had no effect on cell proliferation and drug resistance (Figure S10A,B, Supporting Information). This suggested that a higher‐order structure of *LIST*, rather than some short sequence motifs, might be responsible for its tumor‐promoting function.

**Figure 4:**
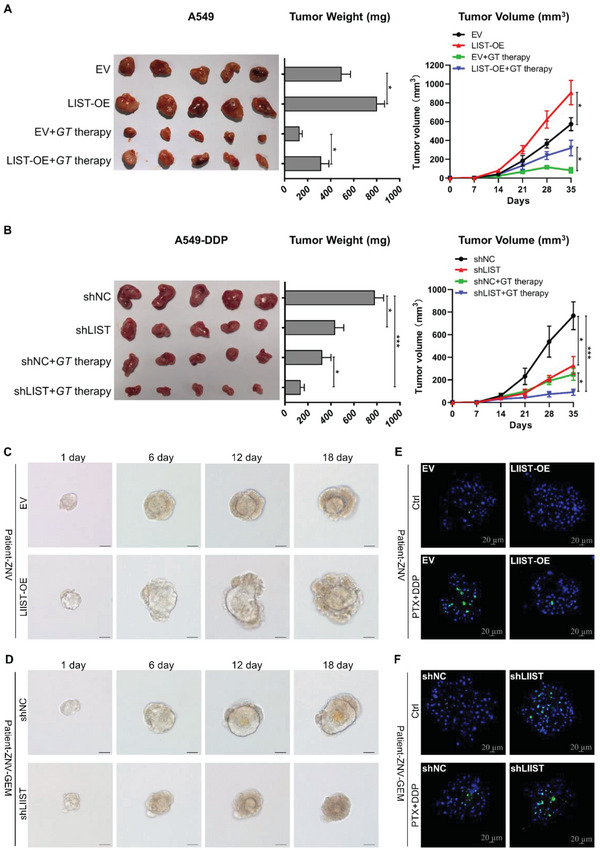
The function of *LIST* in xenograft tumor models and organoid models. A,B) Xenograft tumor models using BALB/c nude mice (*n* = 5) were used to examine the effect of *LIST* A) overexpression or B) knockdown on tumor growth and chemoresistance in vivo. Images, weights, and growth curves of the tumors are presented. *GT* therapy was based on a combination of gemcitabine and paclitaxel. Error bars represent the mean ± SD of the tumors (*p < 0.05, **p < 0.01, ***p < 0.001), Student's *t*‐test. C,D) Organoid growth was examined by measuring organoid volume at multiple time points upon *LIST* overexpression or knockdown. E,F) Apoptotic cells (green) were stained using the caspase 3/7 activity apoptosis assay kit in bladder carcinoma organoid upon *LIST* overexpression or knockdown. PTX (paclitaxel), 0.05µM; DDP (cisplatin), 5µM. Scale bar: 20 µm.

Additionally, multi‐cell lines were used to establish in vivo xenograft tumor models. When compared to control cells, the outcome demonstrated that *LIST* overexpression resulted in a reduction of c‐Src (Y530) in cancer cells (cell lines 5637 and A549) and a significant advantage in tumor growth (**Figure** **4**A, and Figures S11A and S12A, Supporting Information). In contrast, the *LIST* knockdown group had significantly smaller tumor volumes and higher c‐Src (Y530) than the control group (Figure 4B, and Figures S11B and S12B, Supporting Information). Moreover, *LIST* knockdown induced a further deterioration of these parameters in groups treated with chemotherapy (Figure 4B, and Figures S11B and S12B, Supporting Information). In contrast, *LIST* overexpression dramatically attenuated this therapeutic effect (Figure 4A, and Figures S11A and S12A, Supporting Information).

Patient‐derived organoids are a robust preclinical model for investigating sensitivity to therapy. Previously, we developed an organoid model of bladder carcinoma.^[^ ^25^ ^]^ Similar to the cell line results, *LIST* expression was increased during organoid resistance (Figure S13A, Supporting Information). The proliferation assay showed that *LIST* overexpression significantly promoted organoid growth (Figure 4C and Figure S13B, Supporting Information). In contrast, *LIST* knockdown significantly reduced the proliferative capacity of gemcitabine‐resistant organoids (Figure 4D and Figure S13C, Supporting Information). After treatment with chemotherapy drugs, the proportion of apoptotic cells in the *LIST* overexpression group was significantly decreased (Figure 4E and Figure S13D, Supporting Information), while the proportion of apoptotic cells in the *LIST* knockdown group was significantly increased (Figure 4F and Figure S13E, Supporting Information). Furthermore, measuring the IC_50_ values of organoids revealed that *LIST* significantly increased the sensitivity of organoids to multiple chemotherapeutic drugs (Figure S13F,G, Supporting Information). It is abundantly clear from the preceding results that the lncRNA *LIST* has a significant tumor‐promoting effect on the progression and chemoresistance of multiple cancers.

### 2.4 Correlation Analysis of *LIST*, c‐Src, and Clinical Features of NSCLC Tissues

**Figure 5:**
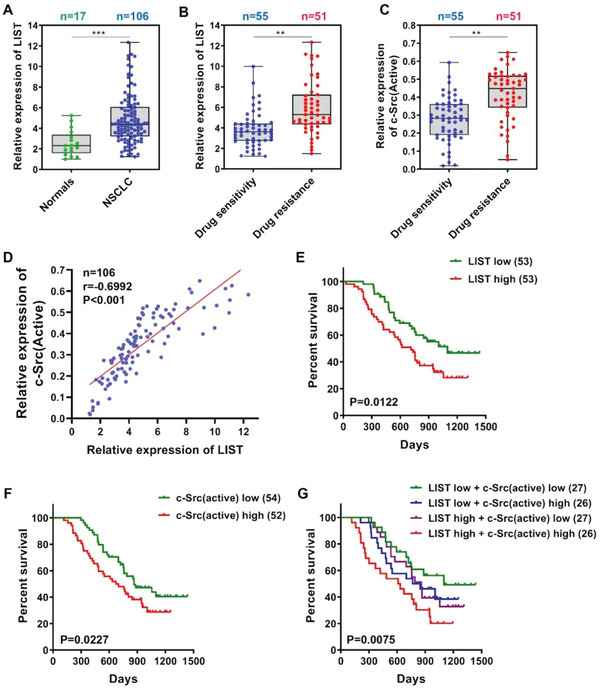
Correlation analysis of *LIST*, c‐Src, and clinical features of NSCLC tissues. A) The expression level of *LIST* in normal tissues and NSCLC tissues was detected via qRT‐PCR. B) NSCLC tissue samples were divided into drug‐sensitive and drug‐resistant groups according to the RECIST 1.1 guidelines, and the difference in *LIST* expression between the two groups was analyzed via qRT‐PCR. C) The differential expression of c‐Src activity between the drug‐sensitive and drug‐resistant groups was assessed. The c‐Src activity was calculated by the following equation: 1 minus p‐c‐Src (Y530)/c‐Src ratio. The abundance of c‐Src and p‐c‐Src (Y530) was determined by western blotting. D). Spearman's correlation was used to analyze the relationship between *LIST* expression and c‐Src activity in NSCLC tissues. E–G) Kaplan–Meier survival curves showing correlations between *LIST*, c‐Src (active), and overall survival in NSCLC tissues. First, these specimens were divided into different subgroups based on their expression levels of E) *LIST* or F) c‐Src (active). G) Next, the specimens were partitioned into four new subgroups based on their cross‐expression (*LIST* low/high and c‐Src (active) low/high). *p*‐values were calculated using the log‐rank test. The error bars represent the mean ± SD (**p < 0.01, ***p < 0.001). Two‐tailed unpaired *t*‐test.

We further investigated the physiological relevance of c‐Src and *LIST* in clinical samples. We first detected *LIST* expression in non‐small cell lung cancer (NSCLC) and normal lung tissues. Consistent with the previous data shown in Figure S3, Supporting Information, *LIST* expression was significantly elevated in NSCLC tissues compared to that in normal lung tissues (**Figure** **5**A). Correlation analyses showed that high *LIST* levels were significantly correlated with TNM classification (Table S1 and Excel S1, Supporting Information). Moreover, NSCLC tissue samples were divided into drug‐sensitivity and drug‐resistance groups according to the Response Evaluation Criteria in Solid Tumors (RECIST 1.1), which indicated that the drug‐resistant group had a higher level of *LIST* expression (Figure 5B and Table S2, Supporting Information). Y530 phosphorylation of c‐Src inactivates its function. Indeed, the phosphorylation level of c‐Src (Y530) was decreased in the chemoresistant cell lines compared with the chemosensitive cells (Figure S14A, Supporting Information). Consistently, the drug‐resistance groups exhibited weaker phosphorylation levels of c‐Src (Y530) than the drug‐sensitivity group (Figure S14B,C, Supporting Information). Therefore, we used the parameter (1 minus p‐c‐Src (Y530)/c‐Src ratio) as an indicator of c‐Src activity, which was also significantly elevated in the drug‐resistant group (Figure 5C and Table S3, Supporting Information). Furthermore, correlation analysis revealed a significant positive correlation between c‐Src activity and *LIST* expression in NSCLC tissues (Figure 5D). Finally, survival analysis was performed to predict the clinical prognosis of patients. The results demonstrated that NSCLC patients with better prognoses had lower expression levels of *LIST* or c‐Src (active) (Figure 5E,F). The patients were divided into four groups based on differences in *LIST* and c‐Src (active) expression. Consequently, the group with lower expression of both *LIST* and c‐Src (active) had the best prognosis, while the group with the highest expression of both factors had the poorest prognosis (Figure 5G). These results suggest that NSCLC progression should correlate with the expression levels of both *LIST* and c‐Src (active), which can be used as prognostic indicators in NSCLC patients.

### 2.5 *LIST* Regulates the Molecular and Biological Functions of c‐Src

**Figure 6:**
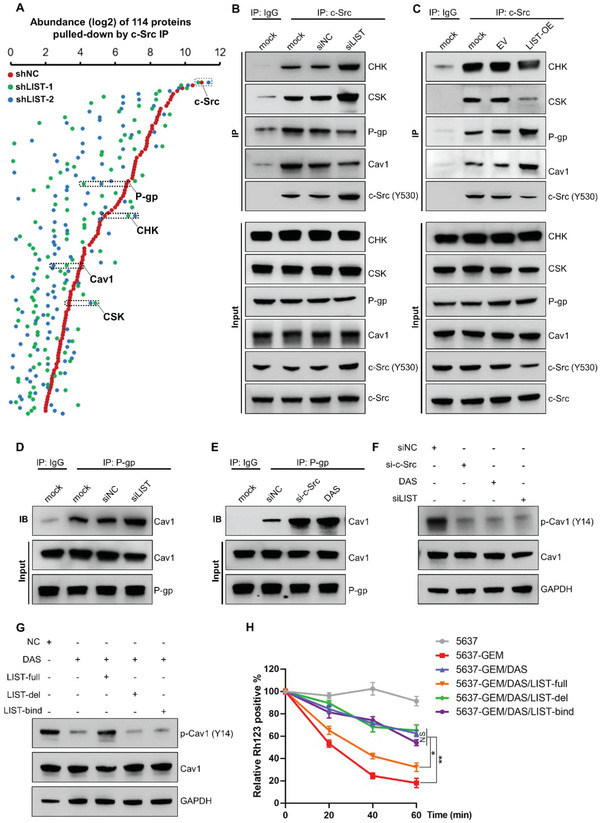
The molecular function of c‐Src was controlled by *LIST*. A) 5637‐GEM cells with *LIST* knockdown were immunoprecipitated for c‐Src, followed by mass spectrometry. As expected, the most abundant protein, c‐Src, was consistently abundant across the groups and served as a positive control. B) c‐Src was immunoprecipitated in *LIST*‐knockdown 5637‐GEM cells, followed by the immunoblotting of CHK, CSK, P‐gp, Cav1, and c‐Src (Y530) proteins. C) c‐Src was immunoprecipitated in *LIST*‐overexpressing 5637 cancer cells, followed by immunoblotting for CHK, CSK, P‐gp, Cav1, and c‐Src (Y530) proteins. D) P‐gp was immunoprecipitated in *LIST*‐knockdown 5637‐GEM cells, followed by the immunoblotting of Cav1 proteins. E) P‐gp immunoprecipitated in c‐Src knockdown or c‐Src inhibitor (DAS) treated 5637‐GEM cells, which was followed by the immunoblotting of Cav1 proteins. F) The total protein and phosphorylation levels of Cav1 were detected by western blotting in different conditions treated 5637‐GEM cells (c‐Src inhibitor, c‐Src knockdown, or *LIST*‐knockdown). G) The total protein and phosphorylation levels of Cav1 in 5637‐GEM cells treated with c‐Src inhibitor were determined by western blotting, and the rescue effect was examined by overexpressing *LIST*‐full or mutants. H) The Rh123 efflux assay was performed in different conditions treating 5637‐GEM cells. Prior to being cultured in a Rh123‐free medium for various amounts of time, cells were initially treated for 30 min in a Rh123 dye‐containing medium. At each time point, the Rh123‐positive cells were immediately detected by using flow cytometry. Cell line 5637 served as a positive control. The error bars represent the SD of three replicates (*p < 0.05, **p < 0.01), Student's *t*‐test.

These results indicate that *LIST* directly binds to c‐Src and regulates its Y530 phosphorylation level. Specifically, the Y530 site of c‐Src is located in the SH1‐C domain, where *LIST* fragment 6 binds. Therefore, we hypothesized that *LIST* could prevent the phosphorylation of Y530 by blocking the binding of c‐Src to upstream kinase proteins. We first performed an immunoprecipitation and mass spectrometry (IP‐MS) assay for c‐Src, which showed that the knockdown of *LIST* altered the c‐Src binding affinity for the vast majority of its binding partners (**Figure** **6**A and Excel S2, Supporting Information). The interactions between protein kinases (CHK and CSK) and c‐Src were largely enhanced upon *LIST* knockdown (Figure 6A). CHK and CSK are known to inhibit c‐Src activity by binding and promoting Y530 phosphorylation.^[^ ^26^, ^27^ ^]^ The Co‐Immunoprecipitation (Co‐IP) results further confirmed that the knockdown of *LIST* increased c‐Src binding to CHK and CSK kinases (Figure 6B), whereas *LIST* overexpression disrupted the binding of c‐Src to CHK and CSK (Figure 6C). This validates our hypothesis that *LIST* inhibits c‐Src Y530 phosphorylation by blocking the binding of c‐Src to kinase proteins.

In addition, the IP assay showed that the interaction of c‐Src with P‐gp and Cav1 was disrupted upon *LIST* knockdown (Figure 6A–C). P‐glycoprotein (P‐gp/MDR1) is a crucial drug pump that efflux drugs out of cells,^[^ ^28^ ^]^ and its transport activity is hindered upon its binding to Cav‐1.^[^ ^29^, ^30^ ^]^ To further validate whether *LIST* mediates the interaction between P‐gp and Cav1, we performed a Co‐IP assay for P‐gp, which showed that the knockdown of *LIST* improved the binding of P‐gp and Cav1 (Figure 6D). Consistently, a Co‐IP assay for P‐gp also confirmed that the interaction between P‐gp and Cav1 was increased in c‐Src knockdown or c‐Src inhibitor‐treated cells (Figure 6E). A previous study reported that phosphorylation of Cav1 decreases its binding to P‐gp and enhances P‐gp activity.^[^ ^31^ ^]^ We examined whether c‐Src regulates the phosphorylation of Cav1 given that it interacts with Cav1 and is a tyrosine‐protein kinase. The outcomes demonstrated that cells treated with c‐Src inhibitors or c‐Src knockdown had lower levels of Cav1 phosphorylation (Figure 6F). The phosphorylation of Cav1 is likewise prevented by *LIST* knockdown (Figure 6F). Furthermore, the decreased phosphorylation of Cav1 brought on by the c‐Src inhibitor can be restored by overexpressing the full‐length *LIST* but not the *LIST* mutant (Figure 6G). Then, we performed a Rh123 efflux assay to examine whether *LIST* regulates P‐gp activity through c‐Src. As shown in Figure 6H, ≈60% of Rh123 dye remained deposited in c‐Src inhibitor‐treated cells, whereas full‐length *LIST* overexpression but not the *LIST* mutant reduced the deposition of Rh123 to ≈30%. Besides, a multidrug efflux transporter P glycoprotein (MDR1/P‐gp) ligand screening assay also showed that intracellular accumulation of fluorogenic P‐gp substrate hydrolysis product was increased in the presence of c‐Src inhibitor (Figure S15A, Supporting Information). Moreover, the full‐length *LIST* can flux P‐gp substrate out of cells while the *LIST* mutant cannot (Figure S15A, Supporting Information). These results demonstrate that *LIST*/c‐Src mediates P‐gp activity by modulating Cav1 phosphorylation, thus leading to increased tumor chemoresistance.

In fact, *LIST* knockdown significantly attenuated the binding between c‐Src and most of the binding proteins (Figure 6A). To obtain more accurate results, we performed an IP‐MS assay targeting c‐Src in cells with *LIST* overexpression. Consistent with previous results, the binding of c‐Src to CHK and CSK was significantly decreased upon *LIST* overexpression, whereas the binding of c‐Src to P‐gp and Cav1 were clearly enhanced (Figure S15B and Excel S3, Supporting Information). Notably, more than half of the c‐Src‐binding proteins overlapped in both the *LIST* overexpression group (53/97) and *LIST* knockdown group (53/114) (Figure S15C, Supporting Information), whose functions were mainly related to cell growth‐associated signaling pathways (Figure S15D, Supporting Information). These data provide high‐throughput corroboration for the potential role of *LIST* in mediating c‐Src molecular functions.

**Figure 7:**
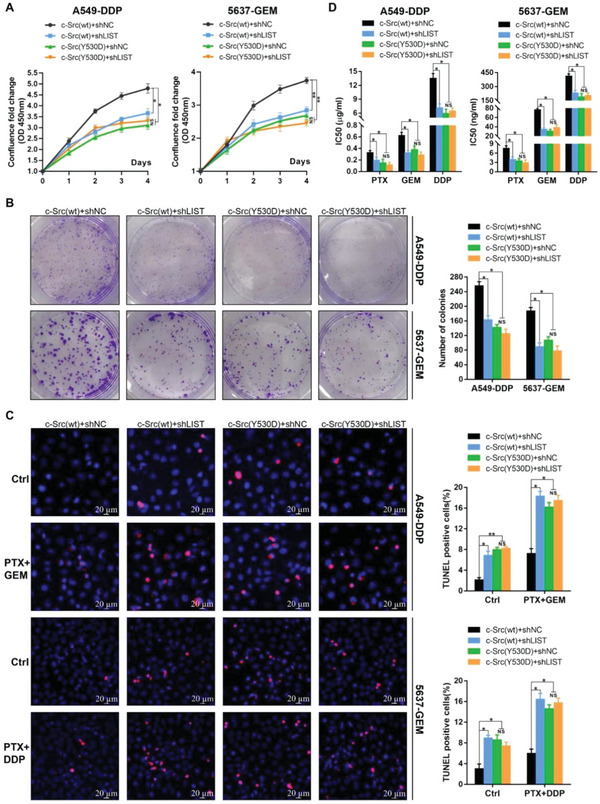
The biological functions of c‐Src were regulated by *LIST*. A–D). c‐Src wild‐type (c‐Src[wt]) or c‐Src mutant (c‐Src[Y530D], Y530 mimics phosphorylation, Y‐D) was reintroduced into c‐Src^−/−^ cells, which were established using the CRISPR/CAS9 system. The proliferation ability of these cells was detected by the A) CCK‐8 assay and B) colony formation assay upon knockdown of *LIST*. C) TUNEL assay was performed to detect apoptosis (red) upon *LIST* knockdown. D) The IC_50_ values of these cells upon *LIST* knockdown were examined using the CellTiter‐Glo Kit. All experiments were performed using A549‐DDP and 5637‐GEM cells. PTX (paclitaxel), 0.05 µM; GEM (gemcitabine), 5 µM; DDP (cisplatin), 5 µM. The error bars represent the SD of three replicates (NS: not significant, *p < 0.05, **p < 0.01), Student's *t*‐test.

It has been reported that the Y530 dephosphorylation of c‐Src is essential for its functional activation. As a result, after gene knockout of c‐Src via Clusterd Regularly Interspaced Short Palindromic Repeats (CRISPR/Cas9）assay, the re‐expression of the c‐Src mutant (Y530 mimics phosphorylation, Y‐D), instead of c‐Src wild type, failed to restore factors related to cell growth ability and chemoresistance, such as cell proliferation (**Figure** **7**A, comparing the black vs green curves), colony formation (Figure 7B, comparing the black vs green bars), cell apoptosis (Figure 7C, comparing the black vs green bars), and cell IC_50_ values (Figure 7D, comparing the black vs green bars). Importantly, upon knockdown of *LIST*, wild‐type c‐Src lost its rescue effect on tumor cell progression and chemoresistance (Figure 7A–D, comparing the blue vs orange groups). The results imply that the biological functions of c‐Src may depend on *LIST*. In addition, we confirmed that the knockdown of *LIST* significantly restrained cell growth and chemoresistance (Figure 7A–D). Specifically, the strong phenotypic consequences of *LIST* only depended on the wild‐type c‐Src protein (Figure 7A–D, comparing the black vs blue groups) but not on the c‐Src^Y530D^ mutant protein (Figure 7A–D, comparing the green vs orange groups). This result indicates that *LIST* promotes tumor chemoresistance and progression via the blocking of c‐Src Y530 phosphorylation.

Additionally, we re‐expressed the c‐Src^Y530A^ mutant (Y530 mimics dephosphorylation; Y‐A) or wild type after the c‐Src gene was knocked out in chemosensitive cells. Figure S16, Supporting Information shows that the c‐Src^Y530A^ mutant exhibited a significant advantage in factors related to cell growth ability and chemoresistance compared with the wild‐type, such as cell proliferation (Figure S16A, Supporting Information, comparing the black vs green curves), colony formation (Figure S16B, Supporting Information, comparing the black vs green bars), cell apoptosis (Figure S16C, Supporting Information, comparing the black vs green bars), and cell IC_50_ values (Figure S16D, Supporting Information, comparing the black and green bars). Interestingly, overexpression of *LIST* lost its tumor‐promoting effect upon re‐expression of the Src^Y530A^ mutant (Figure S16, Supporting Information, comparing the green vs orange groups). This was reasonable because the c‐Src^Y530A^ protein was already in an active state and no longer required the assistance of *LIST*.

In summary, the above results showed that the phosphorylation level and molecular functions of c‐Src in cells were dependent on the lncRNA *LIST*. In turn, *LIST* relies on c‐Src to promote tumor development.

### 2.6 c‐Src/P65 Axis Regulates the Transcriptional Expression of *LIST*

**Figure 8:**
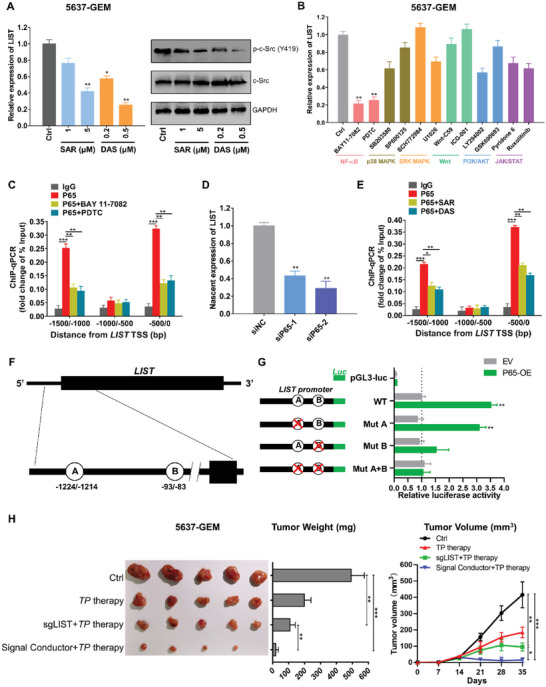
c‐Src regulated the transcriptional expression of *LIST* through NF‐*κ*B pathway. A) Left: Changes in *LIST* expression (left, qRT‐PCR) were determined in 5637‐GEM cells treated with c‐Src inhibitors (dasatinib [DAS] and saracatinib [SAR]). Right: Corresponding c‐Src and P65 phosphorylation levels were measured by western blotting. The error bars represent the SD of three replicates (*p < 0.05, **p < 0.01), Student's *t*‐test. B) The expression level of *LIST* was determined in 5637‐GEM cells treated with various inhibitors of signaling pathways using qRT‐PCR. The error bars represent the SD of three replicates (**p < 0.01), Student's *t*‐test. C) The binding regions of P65 on the *LIST* promoter were identified via ChIP‐qPCR in 5637‐GEM cells; IgG served as a negative control. BAY 11–7082 and PDTC are inhibitors of the NF‐*κ*B pathway The error bars represent the SD of three replicates (**p < 0.01, ***p < 0.001), Student's *t*‐test. D) By capturing nascent RNA, the relative expression levels of the nascent *LIST* transcripts were determined in 5637‐GEM cells with P65 knockdown. The error bars represent the SD of three replicates (**p < 0.01), Student's *t*‐test. E) The binding regions of P65 on the *LIST* promoter were identified via ChIP‐qPCR in 5637‐GEM cells; IgG served as a negative control. Dasatinib (DAS) and Saracatinib (SAR) are c‐Src inhibitors. The error bars represent the SD of three replicates (*p < 0.05, **p < 0.01, ***p < 0.001), Student's *t*‐test. F) Schematic drawing of the potential binding sites for P65 at the *LIST* promoter predicted by the GTRD database. G) The binding sites for P65 at the *LIST* promoter were examined via a dual‐luciferase reporter assay. pGl3‐luc served as a negative control. The data were normalized to renilla luciferase. The error bars represent the SD of three replicates (**p < 0.01), Student's *t*‐test. H) Xenograft tumor models were used to examine the differential effects of CRISPR/Cas9‐based “signal conductors” or *LIST* knockout on tumor growth and chemoresistance in vivo. Images, weights, and growth curves of tumors are presented sequentially from left to right. *TP* therapy was based on a combination of paclitaxel and cisplatin. Error bars represent the mean ± SD of the tumors (*p < 0.05, **p < 0.01, ***p < 0.001), Student's *t*‐test.

Because *LIST* expression was found to increase upon tumor chemoresistance, we investigated the specific mechanism underlying the elevated expression of *LIST* in tumor chemoresistance. We found that inhibition of c‐Src activity (dasatinib and saracatinib served as c‐Src inhibitors) resulted in decreased expression of *LIST* (**Figure** **8**A), suggesting that a positive regulatory loop may exist between c‐Src and *LIST*. To understand the molecular basis underlying c‐Src‐regulated *LIST* expression, we performed a phospho‐array analysis of proteins from 5637‐GEM cells treated with a c‐Src inhibitor. The results revealed that several key signaling molecules were altered as a result of c‐Src activity inhibition; these mainly included enrichment of NF‐*κ*B, p38 MAPK, ERK, Wnt, PI3K/AKT, and JAK/STAT signaling molecules (Figure S17A,B and Table S4, Supporting Information). Further treatment of cells with inhibitors of these signaling pathways revealed that inhibition of only the NF‐*κ*B pathway significantly downregulated the expression of *LIST* (Figure 8B). Given the crosstalk between signaling pathways, it is understandable that these pathways also regulate *LIST* expression to a small extent. We also confirmed that inhibition of c‐Src activity could significantly inhibit the phosphorylation level of P65 (Figure S17C, Supporting Information). A previous study found that P65 phosphorylates in the nucleus and regulates the expression of target genes as a transcription factor when the NF‐*κ*B pathway is activated. Furthermore, the Chromatin immunoprecipitation (ChIP) assay demonstrated that P65 was specifically bound to the promoter region (−1500/−1000 bp and −500/0 bp relative to the transcription start site) of *LIST* (Figure 8C). Specifically, the NF‐*κ*B pathway inhibitors conspicuously reduced the binding capacity of P65 to the *LIST* promoter (Figure 8C). In addition, qPCR analysis confirmed that p65 knockdown significantly inhibited *LIST* expression, whereas overexpression of P65 enhanced *LIST* expression (Figure S18A, Supporting Information). Next, by capturing the uridine analog 5‐ethynyl uridine (EU)‐labeled newly synthesized RNA, we showed that repression of P65 could reduce the nascent *LIST* level (Figure 8D), while P65 overexpression led to elevated levels of nascent *LIST* (Figure S18B, Supporting Information). Notably, the degradation rate of *LIST* was unaffected upon knockdown or overexpression of P65 when treated with actinomycin D (Figure S18C, Supporting Information). Additionally, the ChIP‐qPCR assay also confirmed that c‐Src inhibitors attenuated P65 enrichment at the *LIST* promoter (Figure 8E). Consistently, c‐Src inhibitors could negate *LIST* expression upon overexpression of P65 (Figure S18D, Supporting Information). These results demonstrated that c‐Src positively regulates *LIST* transcription by activating the NF‐*κ*B signaling pathway to recruit the P65 transcription factor.

To explore the mechanism underlying the P65‐mediated upregulation of *LIST*, we first searched for two putative P65 binding sites (−83/−93 and −1214/−1224 bp relative to the transcription start site) in the *LIST* promoter region using the JASPAR database (Figure 8F); these were consistent with the ChIP‐qPCR results. Using a dual‐luciferase reporter system, we then demonstrated that p65 overexpression significantly increased *LIST* promoter activity (Figure 8G). Furthermore, mutating the B binding site (−83/−93 bp) indeed attenuated the P65 effect on *LIST* promoter activity, whereas mutating the A binding site (−1214/−1224 bp) did not affect *LIST* promoter activity (Figure 8G). These results indicate that the −83/−93 bp sites of the *LIST* promoter are the key sites for transcriptional activation of the *LIST* gene via p65.

We have developed a CRISPR/Cas9‐based “signal conductor” that creates a synthetic link to control the transcription of endogenous genes.^[^ ^32^ ^]^ Based on the transcriptional regulation of *LIST* by NF‐*κ*B, we next designed an NF‐*κ*B‐responsive *LIST* gene regulatory element that could recognize and bind to the transcription factor P65 and initiate gene knockout of *LIST* (Figure S19A, Supporting Information). The signal conductor was able to silence *LIST* expression more efficiently than sg*LIST*, especially upon drug treatment (Figure S19B–E, Supporting Information). This was reasonable because cells treated with drugs (such as cisplatin) can activate the NF‐*κ*B pathway, thereby initiating “signal conductor” activity. Moreover, compared with the sg*LIST* group, the mouse xenograft of 5637‐GEM cells with a signal conductor showed significantly reduced tumor growth (Figure 8H and Figure S19F,G, Supporting Information). Taken together, these data suggest that P65 transcriptionally regulates the expression of *LIST* and reveals a positive feedback regulatory loop between c‐Src and *LIST*.

### 2.7 *LIST*/c‐Src Interaction is Associated with Evolutionarily New Variations of c‐Src

As a non‐receptor tyrosine kinase, c‐Src is highly conserved across organisms. However, the sequence of *LIST* is not well conserved (Figure S20A, Supporting Information). Conservation analysis shows that the *LIST* genome sequence is similar in a variety of primates, including chimpanzees, gorillas, and baboons, but not in most other species, including mice and pigs. Therefore, it is worth exploring why c‐Src activity is dependent on *LIST* in human cells but is absent in other species, such as mice.

**Figure 9:**
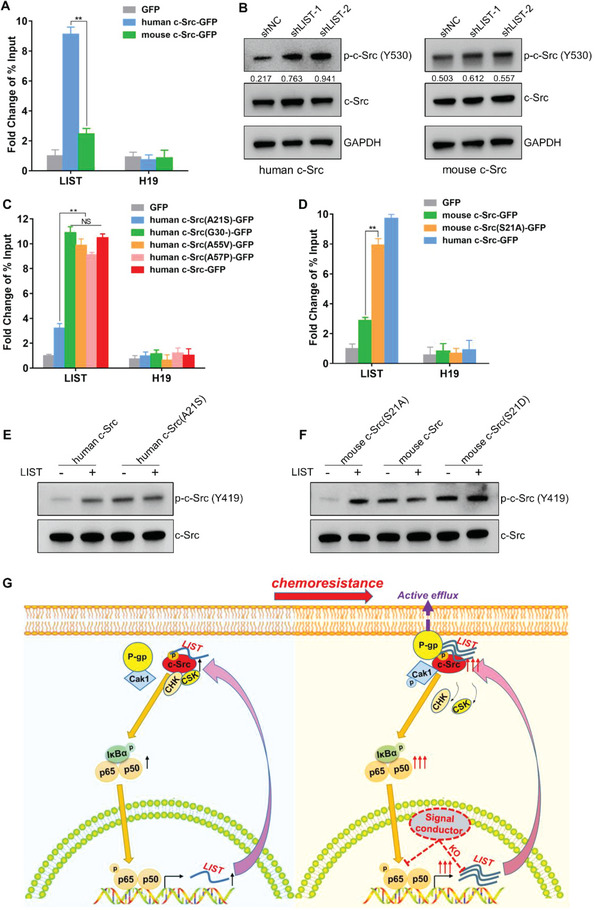
Differences between human and mouse c‐Src in the interactions with *LIST*. A) GFP‐tagged human or mouse c‐Src was re‐expressed into c‐Src^−/−^cell line 5637‐CEM. Enrichment of *LIST* was detected using GFP‐RIP‐qPCR. H19 was used as a negative control. The error bars represent the SD of three replicates (**p < 0.01), Student's *t*‐test. B) Mouse or human c‐Src was re‐expressed into c‐Src^−/−^cell line 5637‐CEM. The total protein and phosphorylation levels of c‐Src were detected by western blotting upon *LIST* knockdown. The proteins were quantified by Image J software. The numbers represent the ratio of p‐c‐Src‐Y530/c‐Src. C) GFP‐tagged wild‐type or mutant human c‐Src at the four residues individually was reintroduced into c‐Src^−/−^cell line 5637‐CEM. Enrichment of *LIST* was detected using GFP‐RIP‐qPCR. H19 was used as a negative control. The error bars represent the ± SD of three biological replicates (NS represents not significant, **p < 0.01), Student's *t*‐test. D) GFP‐tagged wild‐type human c‐Src, mouse c‐Src, or mutant mouse c‐Src at the 21 residues (Ser to Ala) was reintroduced into c‐Src^−/−^cell line 5637‐CEM. Enrichment of *LIST* was detected using GFP‐RIP‐qPCR. H19 was used as a negative control. The error bars represent the ± SD of three biological replicates (**p < 0.01), Student's *t*‐test. E) In vitro phosphorylation assay for purified wild‐type or mutant human c‐Src, which was incubated with *LIST* for 1 h. The phosphorylation state was determined using anti‐c‐Src (phospho Y419), which represents c‐Src activity. F) In vitro phosphorylation assay for purified wild‐type or mutant mouse c‐Src (S21A mimics non‐phosphorylation; S21D mimics phosphorylation), which was incubated with *LIST* for 1 h. The phosphorylation state was determined using anti‐c‐Src (phospho Y419), which represents c‐Src activity. G) Schematic diagram of c‐Src/*LIST* positive feedback loop regulating tumor progression and chemoresistance.

We first re‐expressed mouse or human c‐Src in the c‐Src^−/−^cell line 5637‐CEM to examine its rescue on cell proliferation. Consistently, the rescue function of human c‐Src was always dependent on the expression of *LIST*, whereas mouse c‐Src did not require the assistance of *LIST* (Figure S20B, Supporting Information). Interestingly, RIP assay showed that the binding affinity of mouse c‐Src to *LIST* is much lower than that of human c‐Src, under similar context of c‐Src restoration in the c‐Src^−/−^cell line 5637‐GEM (**Figure** **9**A). Additionally, the phosphorylation level of mouse c‐Src was not affected by *LIST* knockdown (Figure 9B). These results suggest that the human and mouse c‐Src present important differences in terms of their *LIST* dependence.

Then, we aligned human and mouse c‐Src protein sequences and showed that the SH1, SH2, and SH3 domains were exactly the same, while four mutations occurred in the unique domain, which was responsible for binding to *LIST* (Figure S21A, Supporting Information). We mutated each of the four residues of human c‐Src to the mouse version and re‐expressed each in the c‐Src^−/−^cell line 5637‐CEM. RIP assay showed that only mutation of residue 21 of human c‐Src to serine impaired its binding to *LIST* (Figure 9C). In vitro assay also confirmed that human c‐Src^A21S^ mutant (Ala to Ser) and c‐Src^A21D^ mutant (Ala to Asp, mimics phosphorylation) obviously weaken the ability to bind to *LIST* (Figure S21B, Supporting Information). In turn, the conversion of mouse c‐Src to its human homolog on residue 21 largely restored its binding to *LIST* (Figure 9D). Together, these results suggest that the non‐phosphorylation state of residue 21 of c‐Src is predominately responsible for the c‐Src/*LIST* interaction in humans.

Several studies have reported that phosphorylation of the c‐Src amino terminus can activate its own Y419 phosphorylation to promote c‐Src activity.^[^ ^33^, ^34^, ^35^ ^]^ We further determined whether phosphorylation of residue 21 of c‐Src promotes its own activity by in vitro phosphorylation assay. The result showed that *LIST* increased the phosphorylation level at the Y419 site of human c‐Src while the c‐Src^A21S^ mutant did not require the assistance of *LIST* (Figure 9E). Furthermore, mouse c‐Src was very easily phosphorylated, and the addition of *LIST* did not promote its activity (Figure 9F). Note that mutation of the Ser residue 21 to its human homolog (non‐phosphorylated state Ala) reduced its own activity, at which point *LIST* restored its helper capacity (Figure 9F). In contrast, after mutating the Ser residue 21 to Asp (D, mimics phosphorylation), its activity was similar to or even slightly stronger than that of mouse c‐Src (Figure 9F). Therefore, it appears that mouse c‐Src can directly promote its own activity by phosphorylation at position 21 without the assistance of *LIST*. However, to activate Tyr419 phosphorylation and support its own activity, human c‐Src must block phosphorylation at Y530 by binding to *LIST*. By comparing species sequences, it is interesting to note that the non‐phosphorylated state of c‐Src residue 21 is exclusively discovered in primates, which is consistent with the conserved pattern of *LIST* (Figure S22, Supporting Information). It implies that there may be a co‐evolutionary relationship between c‐Src and *LIST* and that *LIST*/c‐Src interaction is linked to the emergence of novel c‐Src variants.

## 3 Discussion

Previous studies on c‐Src inhibitors have mainly focused on traditional small‐molecule inhibitors,^[^ ^8^, ^36^ ^]^ in which tumor cells are often prone to chemoresistance. For example, although the combination of bosutinib (a c‐Src inhibitor) and letrozole elicited a response in the initial stage of the treatment of breast cancer, the cancer cells were prone to develop chemoresistance.^[^ ^37^ ^]^ A similar phenomenon of acquired resistance was seen in clinical trials of the c‐Src inhibitor dasatinib in NSCLC and melanoma.^[^ ^38^, ^39^ ^]^ At present, regulatory strategies targeting lncRNAs have gradually become a widely recognized trend in drug design and development.^[^ ^18^, ^40^, ^41^ ^]^ Interestingly, from the perspective of epigenetic regulation, we screened and identified a novel lncRNA‐*LIST* capable of direct binding and inhibition of c‐Src Tyr530 phosphorylation. c‐Src belongs to the Src family of tyrosine kinases (SFKs) and shares structural homology with other family members, including Fyn, c‐Yes, Lck, and Fgr.^[^ ^42^, ^43^ ^]^ In SFKs, all members have a highly conserved tyrosine residue (Tyr530 in c‐Src) in the tail region that once phosphorylated, remains in an inactive conformation.^[^ ^36^, ^44^ ^]^ However, the N‐terminal unique domain, which is the only non‐conserved region within the kinase family, is specific to c‐Src.^[^ ^45^, ^46^ ^]^ *LIST* binds to the N‐terminal unique domain and the C‐terminal SH1 domain of c‐Src via fragments 1 and 6, respectively. Moreover, the binding capacity of fragment 1 to the c‐Src‐unique domain was much higher than that of fragment 6 to the c‐Src SH1 domain. This indicates that the binding of *LIST* to c‐Src is exclusive and unaffected by other SFK members. Mechanistic studies further revealed that *LIST* prevents the phosphorylation of Y530 by blocking the binding of c‐Src with upstream kinase proteins (CSK and CHK). These results suggest that *LIST*, as a novel c‐Src agonist, has the potential for high efficiency and specificity.

Based on previous evaluations of CPC (Coding Potential Calculator),^[^ ^47^ ^]^ CPAT (Coding Potential Assessment Tool),^[^ ^48^ ^]^ and ribosome profiling data from tumor tissues,^[^ ^49^, ^50^ ^]^ we did not find any evidence to support the idea that *LIST* encodes peptides or proteins. *LIST* primarily serves as a noncoding RNA. Focusing on sequence conservation, we found Ser21 phosphorylation of c‐Src can promote its own activity by activating Tyr419 phosphorylation. It may promote a conformational change to regulate the activity, which is worth investigating in future work. Moreover, we noted that the non‐phosphorylation state of residue 21 of c‐Src is responsible for the c‐Src/*LIST* interaction in humans. From the evolutionary point of view, the variation of the Ser residue site to Ala of c‐Src, which emerged in primates together with the *LIST* sequence, renders an extra layer of control for its activity.

We employed the c‐Src activity level (1 minus p‐c‐Src (Y530)/c‐Src ratio) as a tumor marker for the first time and found that it was not only significantly higher in chemoresistant patients but also positively correlated with a poor prognosis for survival. This is consistent with the findings of *LIST*. In addition, we examined the c‐Src (active)/*LIST* axis as a whole and discovered that it was more successful in predicting the prognosis of NSCLC patients, considering the mutual regulatory interaction between c‐Src and *LIST*. Therefore, we propose that the c‐Src (active)/*LIST* axis, rather than any single indicator, may be the most useful as a predictive biomarker for patients with NSCLC.

In recent years, CRISPR‐Cas9 technology has greatly facilitated precise genome targeting manipulation and has been widely used in cancer therapy. For example, coupling CRISPRa screening with scRNA sequencing revealed T cell activation and states, which could inform the design of immunotherapies.^[^ ^51^ ^]^ The nanoparticles co‐loaded with CRISPR/Cas9 (targeting CDK5 gene) and paclitaxel could effectively inhibit tumor growth.^[^ ^52^ ^]^ The dCasRx‐SINEB2 technology was used for translational control of targeted mRNA by coupling the sgRNA of a catalytically inactive CasRx to an integrated SINEB2 domain of uchl1 lncRNA.^[^ ^53^ ^]^ We verified that the c‐Src/NF‐*κ*B pathway regulates *LIST* expression by activating the transcription factor P65. Based on this, we created an NF‐*κ*B‐responsive *LIST* regulatory element that can monitor the NF‐*κ*B pathway in real‐time while acting as a switch to inhibit *LIST* expression and ultimately prevent the development of drug resistance. This therapeutic approach has the potential to create an effective synergy between gene therapy and chemotherapy, as well as offer new approaches to tumor treatment.

In conclusion, these findings suggest that c‐Src and *LIST* are involved in a positive feedback regulatory loop during tumor resistance. Specifically, the c‐Src/NF‐*κ*B pathway is triggered when tumors gradually develop chemoresistance. P65 is then phosphorylated within the nucleus, where it functions as a transcription factor to promote *LIST* transcription. At this point, the amount of *LIST* in the cell is sufficient to prevent c‐Src Tyr530 phosphorylation by inhibiting the binding of c‐Src to CHK and CSK kinases, resulting in a continuous increase in c‐Src activity, which enhances P‐gp transport activity by modulating Cav1 phosphorylation, thereby leading to drug resistance development and malignant tumor growth (Figure 9G). These results have important implications for our understanding of the mechanism of c‐Src in tumor drug resistance and provide insights for the development of novel c‐Src agonists.

## 4 Experimental Section

### Cell Culture and Treatment

All cell lines were obtained from the American Type Culture Collection (ATCC, Manassas, VA, USA) and the Cell Bank of Type Culture Collection of the Chinese Academy of Sciences (Shanghai, China). RPMI‐1640 medium (Corning, NY, USA) and F‐12K medium (Thermo Fisher Scientific, Waltham, MA, USA) were used to culture 5637 and A549 cells, respectively. Dulbecco's modified Eagle's medium (DMEM; high glucose; Corning) was used to culture A‐375 and HEK293T cells. All the cells were cultured in an incubator (37 °C, 5% CO_2_). For the construction of chemoresistant cell lines, A549‐DDP cells were obtained by culturing in a medium containing 0.2 µM cisplatin, and then the dose was gradually increased to 3 µM until the cells grew normally and stably. 5637‐GEM cells were cultured in a medium with a final concentration of 0.05 µM gemcitabine. A375‐PTX cells were cultured in a medium with a final concentration of 0.01 µM paclitaxel.

### Clinical Tissue Specimen

Clinical tissue specimens (17 normal lung tissues and 106 NSCLC tissues) were provided by the Department of Thoracic Surgery, Cancer Hospital, Chinese Academy of Medical Sciences and Peking Union Medical College, and were collected from November 2016 to February 2019. After diagnosis by pathologists, all specimens were further divided into drug‐resistant and drug‐sensitive groups using the Response Evaluation Criteria in Solid Tumors (RECIST Edition 1.1). The Ethics Committee of the Cancer Hospital, Chinese Academy of Medical Sciences and Peking Union Medical College provided the ethical approval for the study. Additionally, informed written consent was obtained from all participants.

### Organoid Culture

The organoid culture was performed according to the previously described protocol^[^ ^25^ ^]^ with slight modifications. First, the tissues were cut into small pieces of less than 2 mm in size and sequentially incubated with collagenase buffer (5 mg mL^−1^ collagenase II and 10 µM Y‐27632 dihydrochloride), washed with AdDMEM/F12, and incubated with TrypLE Express buffer. The separated cells were then resuspended in cold organoid media containing Matrigel after being filtered using a 70 µm cell filter. Lastly, the Matrigel‐cell mixture (30 µl per droplet for a total of 20 droplets) was added uniformly to 6‐well plates. The organoid medium was changed every 2–3 days. For passaging, the mixture droplets were scraped and incubated with TrypLE buffer to remove Matrigel. The organoids were seeded into 6‐well plates at a 1:3 ratio after centrifugation.

The organoid was cultivated in a medium containing 0.005 M gemcitabine for the production of a chemoresistant organoid, and then the dose was gradually increased to 0.03 M until the organoid could grow regularly and consistently.

Bladder urothelial carcinoma (BLCA) tissues were obtained from patients at Shenzhen Second People's Hospital. The research was conducted in accordance with the guidelines of the Regional Ethics Committees for Research (Research Ethics Committee of Shenzhen Second People's Hospital, No. 20210219002), and informed consent was obtained from all patients.

### Plasmids Construction

For overexpression, Q5 high‐fidelity DNA polymerase (NEB) was used to amplify the CDS of *LIST*, *c‐Src*, and *P65* from genomic DNA. The PCR product was cloned into lentiviral plasmids using *Eco*RI and *Sgr*AI sites and named *LIST*‐OE, c‐Src‐OE, and P65‐OE, respectively.

For the construction of c‐Src full‐length, c‐Src fragments (c‐Src‐1, c‐Src‐2, c‐Src‐3, c‐Src‐4, and c‐Src‐5), and a c‐Src mutant (Y530 was mutated, Y‐A or Y‐D), the corresponding sequence was synthesized and cloned into the HindIII and BamHI sites of the pCMV‐GFP vector.

For the construction of the *LIST*‐binding region and *LIST* del fragment, the corresponding sequence was synthesized and cloned into lentiviral plasmids using EcoRI and SgrAI sites, named *LIST*‐bind and *LIST*‐del, respectively.

For shRNA plasmids, oligonucleotides targeting *LIST* were designed, synthesized, and cloned into the pLKO.1‐U6‐shRNA‐EF1a‐GFP‐T2A‐puro vectors, named sh*LIST*‐1 and sh*LIST*‐2.

### Lentivirus Infection

The lentiviral expression vector and packaging plasmids (pLP1, pLP2, and pLP‐VSVG) were cotransfected into HEK293T cells at a 3:2:3:4 ratio using Lipofectamine 2000 (Invitrogen, Waltham, MA, USA). Polybrene (8 µg ml^−1^) was then added to the cells. The cells were centrifuged (2000 rpm for 60 min at 37 °C) and incubated for 48 h. Finally, puromycin was added at the corresponding lethal concentration for screening purposes. The targeting sequence of shRNA was shown in Table S5, Supporting Information).

### siRNA Transfection

Ribobio (Guangzhou, China) provided siRNAs that specifically targeted *LIST* and P65. Lipofectamine RNAiMAX Reagent (Invitrogen) was used to transfect siRNA into cells in accordance with the manufacturer's instructions. The targeting sequence of siRNA is shown in Table S6, Supporting Information.

### Gene Knockout via CRISPR/Cas9

The CRISPR Design Tool (http://crispr.mit.edu/) was used to design and generate sgRNAs targeting c‐Src and *LIST*. Briefly, a pair of sgRNAs targeting c‐Src was inserted into the LentiCRISPRv2 plasmid (Addgene, cat. 52961, Watertown, MA, USA). For CRISPR/Cas9‐based “signal conductors”, the Tet‐on CRISPR/Cas9 system was used. The regulatory element was first constructed by adding the p65 aptamer sequence to the 3ʹ end of the gRNA sequence of *LIST*. This regulatory element was inserted into the Tet‐on pLenti‐sgRNA plasmid (Addgene, cat. 71409). The pCW‐Cas9 plasmid (Addgene ID: 50661) was used to express the Tet‐on Cas9.

For lentivirus packaging, the packaging plasmids (psPAX2 and pVSVG) and lentiviral expression vector were co‐transfected into HEK293T cells using Lipofectamine 2000 (Invitrogen) for two days. The viral supernatant was collected and concentrated to infect the target cells. After the cells were screened using antibiotics, genomic DNA was extracted for genotyping validation and knockout efficiency detection using qRT‐PCR. The sequence of sgRNA is shown in Table S7, Supporting Information. The sequence of regulatory elements for “signal conductor” is shown in Table S8, Supporting Information.

### qRT‐PCR Assay

For qRT‐PCR, RNA was first extracted using a TRIzol reagent. The High‐Capacity cDNA Reverse Transcription Kit (4368814, Invitrogen) was used to synthesize first‐strand cDNA. SYBR Green Master MIX (Invitrogen) was used in the qPCR assay, in accordance with the manufacturer's protocol. The housekeeping gene *GAPDH* was selected as the reference gene for the quantification of target genes. The primer sequences used are shown in Table S9, Supporting Information.

### Measurement of LIST RNA Copy Numbers

RNA copy numbers were estimated as previously described^[^ ^54^ ^]^ with slight modifications. TRIzol reagent was used to extract RNA from 50 000 cells. A standard curve was generated by qRT‐PCR analysis after the RNA samples were subjected to a series of concentration gradients of RNA standards (RNA spike‐in). Finally, the Ct value of the standard curve was used to calculate the number of *LIST* copies in each cell.

### RNA Isolation from Cytoplasm and Nucleus

First, the cells (5 × 10^5^) were centrifuged to obtain the supernatant. The pellet was lysed with 250 µl lysis buffer (1.5 mM MgCl_2_, 140 mM NaCl, 50 mM Tris pH 8.0, 2 mM RVC [(ribonucleoside vanadyl complex], 0.5% NP‐40). Next, the cell sample was incubated on a shaker at 4 °C for 10 min, and 1/10 volume of the sample was aspirated for total RNA extraction. The remaining lysate was centrifuged at 4 °C (1000 rpm, 3 min). The cytoplasmic fraction was in the supernatant, while the nuclei were in the pellet. The nuclear fraction pellets were given three gentle washes with lysis buffer (without NP‐40). Finally, RNA was extracted separately from the two fractions.

### Nascent RNA Capture and RNA Degradation Dynamics Analysis

A day after being seeded in 12‐well plates, the cells were incubated for 24 h with 0.2 mM EU to label newly synthesized nascent RNAs. Finally, using the Click‐iT Nascent RNA Capture Kit (MP10365, Invitrogen), the EU‐labeled RNAs were biotinylated and captured in accordance with the manufacturer's protocol.

Prior to RNA degradation analysis, the cells were treated with different types of gene perturbations. The following day, actinomycin D (5 µM) was added to the cells at different time points. Then, RNA was extracted from the cells to measure *LIST* expression levels by qPCR. Finally, RNA values at different time points were plotted as degradation curves.

### Cell Counting Kit (CCK‐8) Assay and Colony Formation Assay

For the CCK‐8 assay, 5 × 10^3^ cells with different types of gene perturbations were seeded into a 96‐well plate. The CCK‐8 kit was used the following day to measure cell viability for four consecutive days. The cell proliferation curve was made by the OD values at different time points relative to time 0. The OD value at time 0 is normalized to 1.

For colony formation assays, gene perturbation‐treated cells (1 × 10^3^) were seeded into 6‐well plates and cultured for ≈14 days. The medium was changed twice a week. The cells were fixed for 15 min with methanol, washed twice with PBS for 5 min, and stained for 15 min with crystal violet. Finally, the colonies were viewed and counted using ImageJ software.

### Cell Apoptosis Assay

The cells were spread onto cell slides a day in advance and sequentially fixed with 4% paraformaldehyde (25 min), washed three times with PBS, and permeabilized with 1% Triton X‐100 (5 min). Red fluorescein (TRITC) was then labeled to the broken DNA in apoptotic cells for fluorescence microscopy detection using the TUNEL detection kit for cell apoptosis (KGA7062, KeyGen BioTECH), following the manufacturer's protocol. Normal or proliferating cells were rarely labeled because there were few breaks in their DNA.

### Drug IC_50_ Assay

Gene perturbation‐treated cells (5 × 10^3^) were seeded into 96‐well plates a day in advance, and then a series of concentration gradients of chemotherapy drugs were added to the cell medium for three days. The CellTiter‐Glo reagent (G7570, Promega, Madison, WV, USA) was used to measure cell viability. Finally, cell activity was determined by detecting the fluorescence signal using a microplate reader. The IC_50_ values were calculated by drawing a line graph (abscissa: drug concentration; ordinate: cell viability). Cell viability in the control group was normalized for days 0 to 1.

### Rh123 Efflux Assay

Gene perturbation‐treated cells (1 × 10^6^) were first digested with trypsin, washed with PBS, and incubated in a cell medium containing Rh123 for 30 min for adequate Rh123 uptake. The cells were then centrifuged to remove Rh123, washed three times with PBS, and incubated in a medium without Rh123 for 20, 40, and 60 min, respectively. Finally, the cells were collected at different time points, and flow cytometry or fluorescence microscopy was used to determine the percentage of Rh123‐positive cells. (Ex/Em = 488/530 nm).

### Multidrug Efflux Transporter P Glycoprotein Ligand Screening Assay

A multidrug efflux transporter p glycoprotein ligand screening kit (ab284553, abcam) was used to determine P‐gp efflux activity. Briefly, the cells were seeded in a white‐walled 96‐well plate one day in advance. The next day, the cell medium was replaced with 100 µl fresh efflux assay buffer and the test compound solutions (DAS and verapamil) were added to the cells. Finally, 50 µl of fluorescent P‐gp substrate was added to each well and kept in the dark at 37 °C for 30 min. Spectrofluorometer was used to measure the fluorescence intensity (Ex/Em = 488/532 nm) of all of the wells. For P‐gp efflux activity, it was calculated by the following equation: P‐gp efflux activity = 100 − [(Fr − Fc)/(Fm − Fc)] × •100, where *Fc* is the fluorescence intensity of the no inhibition control condition (control group), *Fm* is the fluorescence intensity of verapamil (positive control), and *Fr* is the fluorescence intensity of the cells under different conditions.

### Western Blotting

Cells were first lysed to extract proteins using RIPA buffer. For tumor tissue samples, the tissues were cut into pieces, added with cold PBS, homogenized with a tissue homogenizer until there was no obvious macroscopic solid, left on ice for 5 min, and carefully aspirated the supernatant into another pre‐cooled clean centrifuge tube. The cells were centrifuged at 500× g for 2–3 min at 4 °C, the supernatant was discarded, and then the RIPA lysate was added. The BCA protein assay kit (23227, Thermo Fisher) was used for protein quantification. The proteins were then dissolved in 1× SDS buffer for heat‐denaturation (100 °C, 10 min) to prepare the final sample. Next, the sample was loaded, transferred to a membrane, blocked with 5% BSA, incubated with the primary antibody, washed with TBST, incubated with secondary antibodies, and washed again with TBST. Bio‐Rad (Hercules, CA, USA) was used to visualize and measure the protein signals. Anti‐c‐Src (1:3000, ab109381), anti‐c‐Src (phospho Y530) (1:1000, ab32078), anti‐c‐Src (phospho Y419) (1:1000, ab133460), anti‐GFP (1:4000, ab290), anti‐P65 (1:3000, ab16502), anti‐P65 (phospho S536) (1:1000, ab76302), anti‐CSK (1:1000, CST, 4980), anti‐CHK (1:1000, CST, 2360), anti‐Cav1 (phospho Y14) (1:1000, CST, 3251), anti‐Cav1 (1:1000, ab2910), anti‐P‐gp (1:1000, ab129450) and anti‐GAPDH (1:4000, ab8245) were purchased from Abcam (Cambridge, UK) and Cell Signaling Technology (Danvers, MA, USA).

### RNA Binding Protein Immunoprecipitation Assays (RIP)

RIP was performed as previously described^[^ ^55^ ^]^ with slight modifications. After harvesting, the gene perturbation‐treated cells (5 million) were lysed using lysis buffer (200 mM NaCl, 100 mM pH 7.4 Tris, 0.5% NP‐40, 2 mM RVC, RNasin, protease inhibitor cocktail (Roche, Basel, Switzerland), 0.5 mM PMSF) and subjected to sonication (10 s for sonication and 50 s for rest, four cycles, power 10). Dynabeads Protein G (10 µL) was added to the sample to remove non‐specific binding. Dynabeads Protein G (25 µL) and the specific antibody (3.8 µg) were mixed simultaneously and incubated on a rotating shaker (2 h at 4 °C). The sample was then added to a mixture of antibody beads and incubated overnight at 4 °C. The following day, wash buffer I (150 mM Tris pH 7.4, 0.1% sodium deoxycholate, 150 mM NaCl, 0.5 mM PMSF, protease inhibitor cocktail, 0.5% NP‐40, 2 mM RVC) and wash buffer II (200 mM Tris pH7.4, 0.1% sodium deoxycholate, 300 mM NaCl, 0.5% NP‐40, protease inhibitor cocktail, 0.5 mM PMSF, 2 mM RVC) were used to remove non‐specific binding (three times, 5 min each). The sample was then treated for 2 h at 55 °C with proteinase K buffer (18 µL proteinase K (10 mg ml^−1^), 15 µL 10% SDS, and 117 µL wash buffer I). The RNA was purified and then used for qRT‐PCR and RNA sequencing analysis.

### RNA Fluorescence In Situ Hybridization Assay (FISH)

The probe for the RNA‐FISH assay was prepared according to the previously described protocol.^[^ ^56^ ^]^ After synthesis, the *LIST* fragment was cloned into a pGEM‐T vector. Finally, the Biotin RNA Labeling Mix (Roche) was used to produce the biotin‐labeled probe.

The FISH assay was performed as previously described.^[^ ^54^ ^]^ The gene perturbation‐treated cells were first spread onto cell slides a day in advance. The cells were fixed the following day for 15 min at room temperature with 4% paraformaldehyde, washed three times with PBS, 0.5% Triton X‐100 for permeability, washed with PBS (three times, 5 min), pre‐hybridized (50% Formamide, Denhardt, 0.01% Tween‐20, 2 × SSC, 10 mM EDTA) for 2 h at 56 °C, and probe hybridization (overnight, 56 °C). On the third day, 3% BSA was used to block the cells, which were treated with specific antibodies: anti‐biotin (1:300, ab23284), anti‐c‐Src (1:500, ab231081), fluorescent secondary antibodies (ab150116, 1:500; ab150077, 1:500), and then stained with DAPI. Finally, a laser‐scanning confocal microscope was used to capture cell images. For tissue in situ hybridization, tissue sections were sequentially deparaffinized in xylene, antigen repaired, permeabilized in acetic acid, dehydrated in ethanol, and then pre‐hybridized.

The Pearson correlation coefficient (PCC) method was used to quantitatively analyze the co‐localization of fluorescence signals in each cell. A maximum PCC value (1.0) indicated complete co‐localization of the two fluorescent signals within the cell, whereas a PCC = 0 indicated no co‐localization. In particular, analysis was carried out in accordance with the software's instructions utilizing NIS‐ElemS‐AR. For each condition, 10 cells were chosen at random, and the PCC values of those cells were recorded.

### RNA (Biotin Label) Pull‐Down Assay

Biotin RNA labeling mix (Roche) was used to transcribe biotin‐labeled *LIST* RNA truncations in vitro. First, Dynabeads MyOne^TM^ Streptavidin C1 (20 µl) were mixed with biotin‐labeled RNA for 2 h at 4°C. The cells (5 million) were lysed with lysis buffer (2 mM RVC, 150 mM NaCl, 1% NP‐40, RNasin, 50 mM Tris pH 7.4, protease inhibitor cocktail, 0.5 mM PMSF), sonicated (10 s for sonication and 50 s for rest, four cycles, power 10), and centrifuged to collect the supernatant. A 1/15 volume of the supernatant was aspirated and stored as an input. The remaining cell supernatant was then mixed with RNA‐bead slurry and stored at 4 °C overnight. The sample was rinsed five times the following day with buffer I (50 mM Tris pH 7.4, protease inhibitor cocktail, 350 mM NaCl, RNasin, 1% NP‐40, 2 mM RVC, 0.5 mM PMSF). Following the heat denaturation in 1 × SDS loading buffer, the sample was subjected to western blotting.

For the in vitro RNA‐protein collaboration examination, the methodology was similar. Briefly, the *LIST* RNA transcript was incubated with Dynabeads (MyOne Streptavidin C1) for 2 h at 4 °C. The RNA‐bead slurry was then mixed with purified protein (c‐Src or c‐Src truncations) in binding buffer (150 mM NaCl, RNasin, protease inhibitor cocktail, 2 mM RVC, 50 mM Tris pH 7.4, 0.5% NP‐40, and 0.5 mM PMSF) at 4 °C overnight. A 1/15 volume of the sample was aspirated and stored as an input. The next day, the samples were washed and denatured for protein detection by western blotting.

### RNA EMSA Assay

A LightShift Chemiluminescent RNA EMSA Kit (20158, Thermo Fisher) was used to perform the RNA EMSA assay. Briefly, the biotin‐labeled *LIST* fragment was incubated with purified c‐Src protein in binding buffer. The samples were then sequentially subjected to native gel electrophoresis, transferred to a nylon membrane, cross‐linked, and streptavidin‐horseradish peroxidase‐conjugated according to the manufacturer's instructions.

### Immunohistochemistry

Paraffin sections were first deparaffinized with xylene and then sequentially subjected to antigen repair in citrate buffer, peroxidase blocking, serum blocking, and overnight incubation with primary antibodies (Anti‐c‐Src [1:400, ab231081], anti‐c‐Src [phospho Y530] [1:200, ab32078], and anti‐c‐Src [phospho Y419] [1:200, ab40660]). The next day, slides were washed three times with PBS and incubated with fluorescent secondary antibodies (Alexa Fluor 488 [1:500, ab150331], Alexa Fluor 594 [1:500, ab150080]) for 1 h at room temperature. Finally, the nuclei were counterstained with DAPI. Immunofluorescence imaging was performed using a ZEISS confocal microscope.

### Phospho Explorer Antibody Microarray Analysis

Dasatinib (0.5 µM) was applied to the cells, and the Phospho Explorer antibody microarray (Full Moon Biosystems, Sunnyvale, CA, USA), which contained 16 signaling pathways and 304 antibodies, was used to analyze the results. An established methodology was followed during the antibody array experiments.

### In Vitro Phosphorylation

The *LIST* transcript was synthesized with a biotin RNA labeling mix (Roche) in vitro. The purified c‐Src‐GFP protein or mutant was incubated with *LIST* transcript in phosphorylation buffer (150 mM NaCl, 10 mM MgCl_2_, 50 mM Tris, pH 7.4, 10 mM MnCl_2_, 1 mM DTT and 50 µM ATP) for 1 h at room temperature. Then SDS solution was added to terminate the phosphorylation reaction. Lastly, the sample was detected by immunoblotting using Anti‐c‐Src, anti‐c‐Src (phospho Y419), or anti‐GFP.

### Dual‐Luciferase Reporter Assay

First, *LIST* promoter sequences (wild‐type or mutant) were synthesized. These sequences were then separately inserted in front of the fluorescent sequences of the pGL3 plasmid to generate the pGL3‐WT and pGL3‐mut plasmids. For the *LIST* promoter luciferase assay, luciferase reporter constructs (pGL3 plasmid and Renilla vector) were transfected into cells treated with gene perturbation. Following the manufacturer's instructions, the Dual‐Glo Luciferase Assay System (Promega) was used to measure the luciferase activity the following day, and Renilla was used as the input.

### Chromatin Immunoprecipitation Assay (ChIP)

The ChIP assay was performed as previously described^[^ ^54^ ^]^ with slight modifications. Briefly, cells (10 million) were first treated with 1% formaldehyde (10 min at 37 °C) and quenched with glycine (0.14 M) for 30 min at room temperature. The lysate (150 mM NaCl, protease inhibitor cocktail, 100 mM Tris pH 7.4, 2 mM RVC, 1% NP‐40, 0.5 mM PMSF) was then added to the cells, followed by sonication to get ≈400–700 bp DNA fragments. The samples were mixed with Dynabeads Protein G for 1 h at 4 °C to remove non‐specific binding (1 h, 4 °C). Meanwhile, 1/15 volume samples were saved as a reference for the input DNA. The anti‐P65 antibody (ab16502, Abcam) or anti‐IgG antibody (ab172730, Abcam) was mixed with the remaining samples and incubated at 4 °C overnight. The following day, the samples were washed with buffer (six times, 5 min), eluted with elution buffer, reverse cross‐linked with proteinase K buffer (55 °C, 2 h), and DNA was purified. Finally, qPCR analysis of the enriched DNA was performed. The primer sequences used were shown in Table S9, Supporting Information.

### Immunoprecipitation and Mass Spectrometry

The gene perturbation‐treated cells (5 million) were lysed with 500 ml of lysis buffer (87787, Thermo; 0.5 mM PMSF; and protease inhibitor cocktail, Roche). Similar to the RIP assay, the samples were also pre‐cleared with Dynabeads Protein G, and 1/15 of them were saved as a reference for input. Meanwhile, Dynabeads Protein G was mixed with a specific antibody (c‐Src or IgG) on a shaker for 2 h (4 °C). After that, the samples were incubated overnight in the antibody‐bead slurry. Both buffer I (50 mM Tris pH 7.4, 2 mM RVC, 150 mM NaCl, 0.5 mM PMSF, 0.5% NP‐40, protease inhibitor cocktail; three times, 5 min) and buffer II (50 mM Tris pH7.4, 2 mM RVC, 0.5 mM PMSF, 350 mM NaCl, 1% NP‐40, protease inhibitor cocktail; three times, 5 min) were used to wash the samples the following day. Western blotting or subsequent mass spectrometry analysis was performed to examine the final samples.

For mass spectrometry analysis, the proteins were separated by SDS‐PAGE, and the ProteoSilver Kit (Sigma‐Aldrich, St. Louis, Mo, USA) was used for silver staining. Finally, the bands were collected for mass spectrometry.

### Animal Experiments

An animal model using BALB/c nude mice aged 4–6 weeks was developed. The gene perturbation‐treated cells (3 million) were harvested and dissolved in PBS (100 µl). The cells were then injected subcutaneously into the right flanks of the mice. In the medical‐therapy group, drugs were intraperitoneally (i.p.) injected after ≈10 days, when the tumor mass was ≈5 mm in diameter. Weekly cycles of cisplatin (DDP, 3 mg kg^−1^, on day 1), gemcitabine (GEM, 1 mg kg^−1^, on days 1–3), and paclitaxel (PTX, 0.4 mg kg^−1^, on days 1–3) were administered. Every seven days, the tumor volume (volume = (length × width^2^)/2) was measured. At the end of the experiment, the tumors were weighed. All mouse experiments were approved by the Institutional Animal Care and Use Committee of Shenzhen University (20220062).

For Tet‐on CRISPR/Cas9‐based “signal conductors”, doxycycline buffer (2 mg mL^−1^ doxycycline and 5% sucrose) was added to the mice's drinking water two weeks after the cells were subcutaneously injected.

### RNA‐Ligase‐Mediated Rapid Amplification of cDNA Ends (RACE) Assay

The SMARTer RACE 5ʹ/3ʹ Kit (634858, Takara, Kusatsu, Japan) was used to perform 5ʹ and 3ʹ RACE assay, in accordance with the manufacturer's instructions. The 5ʹ‐RACE (5ʹ‐TCACCATTACCGCTACATAGTACG3ʹ) and 3ʹ‐RACE (5ʹ‐ GAGACATGCCACCATATTTAGG3ʹ) gene‐specific primers were developed.

### Overall Survival Analysis

Clinical data were obtained from NSCLC tissues from the Department of Thoracic Surgery, the Cancer Hospital, the Chinese Academy of Medical Sciences, and Peking Union Medical College. First, the specimens were divided into different subgroups according to differences in gene expression. The prognosis of these subgroups was then analyzed using patient Kaplan–Meier survival curves.

### *LIST* Expression Analysis from TCGA

*LIST* expression analysis was performed as described previously.^[^ ^55^ ^]^ The RNA‐seq V2 data of 22 cancer specimens from TCGA were used to analyze *LIST* expression levels. All specimens were processed and normalized using upper quantile normalization method.

### Statistical Analysis

Unless otherwise stated, data were analyzed using Student's *t*‐test in Excel 2016 and GraphPad Prism 8. Statistical significance was set at *p* < 0.05. *p < 0.05, **p < 0.01, ***p < 0.001, and NS (not significant). The SD of at least three biological replicates is depicted in the graph as error bars.

## Conflict of Interest

The authors declare no conflict of interest.

## Author Contributions

X.T.W., B.W., F.L., X.K.L., and T.G. contributed equally to this study. X.T.W., B.W., F.L., X.K.L., W.R.H., and T.G. conducted experiments and wrote the manuscript. D.W.W. and S.Y.G. prepared and processed the clinical samples. W.R.H., and X.T.W. conceived of the study. W.R.H., and X.T.W. supervised the study and revised the manuscript. All authors have read and approved the final manuscript.

## Supporting information

Supporting Information

Supporting Information

## Data Availability

The data that support the findings of this study are available from the corresponding author upon reasonable request.
