# The c‐Src/LIST Positive Feedback Loop Sustains Tumor Progression and Chemoresistance

**DOI:** 10.1002/advs.202307933

**Published:** 2023-12-28

**Authors:** Xianteng Wang, Bing Wang, Fang Li, Xingkai Li, Ting Guo, Yushun Gao, Dawei Wang, Weiren Huang

*Adv. Sci*. **2023**, *10*, 2300115

https://doi.org/10.1002/advs.202300115

In the original published article, there are errors in Figures 7, S12 and S16C. Please find below the correct figures. These errors do not affect the results or conclusions of this article. The authors apologize for any inconvenience this may have caused.

**Figure 7:**
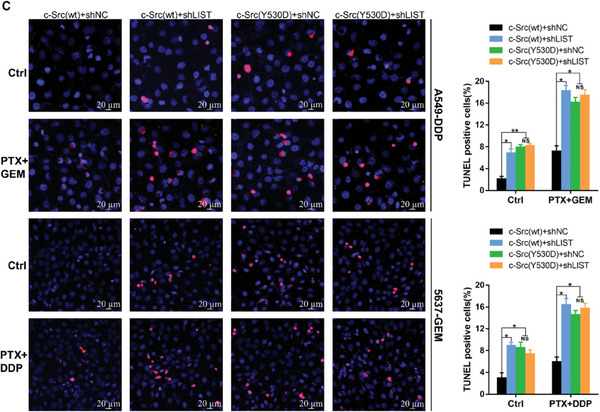
The biological functions of c‐Src were regulated by LIST. (C) c‐Src wild‐type (c‐Src(wt)) or c‐Src mutant (c‐Src(Y530D), Y530 mimics phosphorylation, Y‐D) was reintroduced into c‐Src^−/−^ cells, which were established using the CRISPR/CAS9 system. TUNEL assay was performed to detect apoptosis (red) upon LIST knockdown.

**Figure S12:**
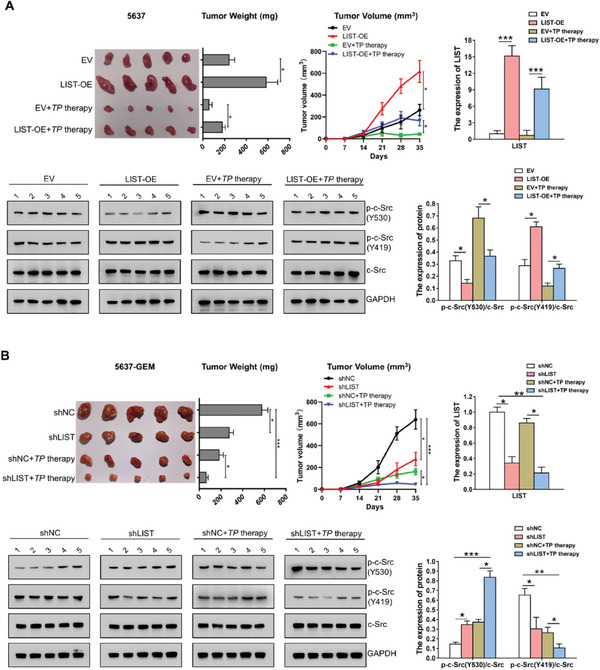
The function of LIST in xenograft tumor models. (A‐B). The impact of LIST overexpression (A) and knockdown (B) on tumor growth and chemoresistance in vivo was investigated using xenograft tumor models. The tumor images, weights, and growth trajectories are displayed. Cisplatin and paclitaxel are used in combination therapy, known as TP therapy. The expression levels of c‐Src(Y530, Y419) and LIST in xenograft tumor models were examined via western blotting or qRT‐PCR. The proteins were quantified by Image J software. The ratio of p‐c‐Src‐Y530/c‐Src or p‐c‐Src‐Y419/c‐Src was analyzed using GraphPad Prism 8. Error bars represent the mean ± SD of the tumors. (A‐B). Error bars show the standard deviation (SD) of five replicates (*P‐value < 0.05, **P‐value < 0.01, ***P‐value < 0.001), Student's t‐test.

**Figure S16:**
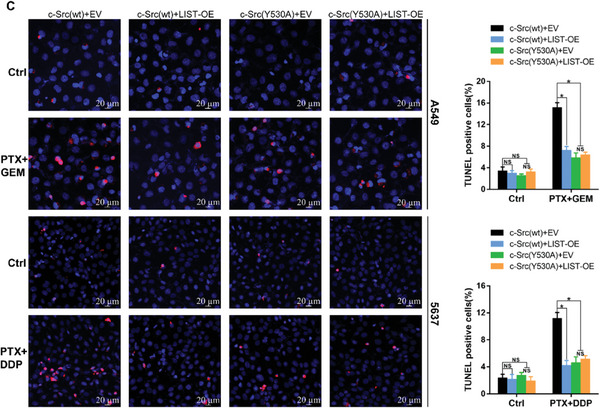
The biological functions of c‐Src were regulated by LIST. (C) In c‐Src^−/−^ cells created using the CRISPR/CAS9 system, c‐Src wild‐type (c‐Src(wt)) or c‐Src mutant (c‐Src(Y530A), where Y530 mimics dephosphorylation, Y‐A) was reintroduced. The TUNEL assay was used to examine apoptotic cells (red) in response to LIST overexpression.

