# Supplementary material for: The c‐Src/LIST Positive Feedback Loop Sustains Tumor Progression and Chemoresistance
Source: Adv Sci (Weinh). 2023 May 8;10(20):2300115. doi: 10.1002/advs.202300115 (PMC10369257; doi:10.1002/advs.202300115)
Supplement: Supplementary file 1 — Supporting Information [file ADVS-10-2300115-s001.pdf]

ADVANCED SCIENCE
Open Access

Supporting Information

for *Adv. Sci.*, DOI 10.1002/advs.202300115

The c-Src/LIST Positive Feedback Loop Sustains Tumor Progression and Chemoresistance

Xianteng Wang, Bing Wang, Fang Li, Xingkai Li, Ting Guo, Yushun Gao, Dawei Wang and Weiren Huang**

Supplementary Materials for

The c-Src/*LIST* positive feedback loop sustains tumor progression and chemoresistance

Xianteng Wang *et al.*

*Corresponding authors. Email: Xianteng Wang (wxt15@tsinghua.org.cn); Bing Wang (bing_wang70@sina.com); Weiren Huang (wr.huang@siat.ac.cn)

This PDF file includes:

Supplementary Fig. S1-S22; Supplementary Table S1-S9

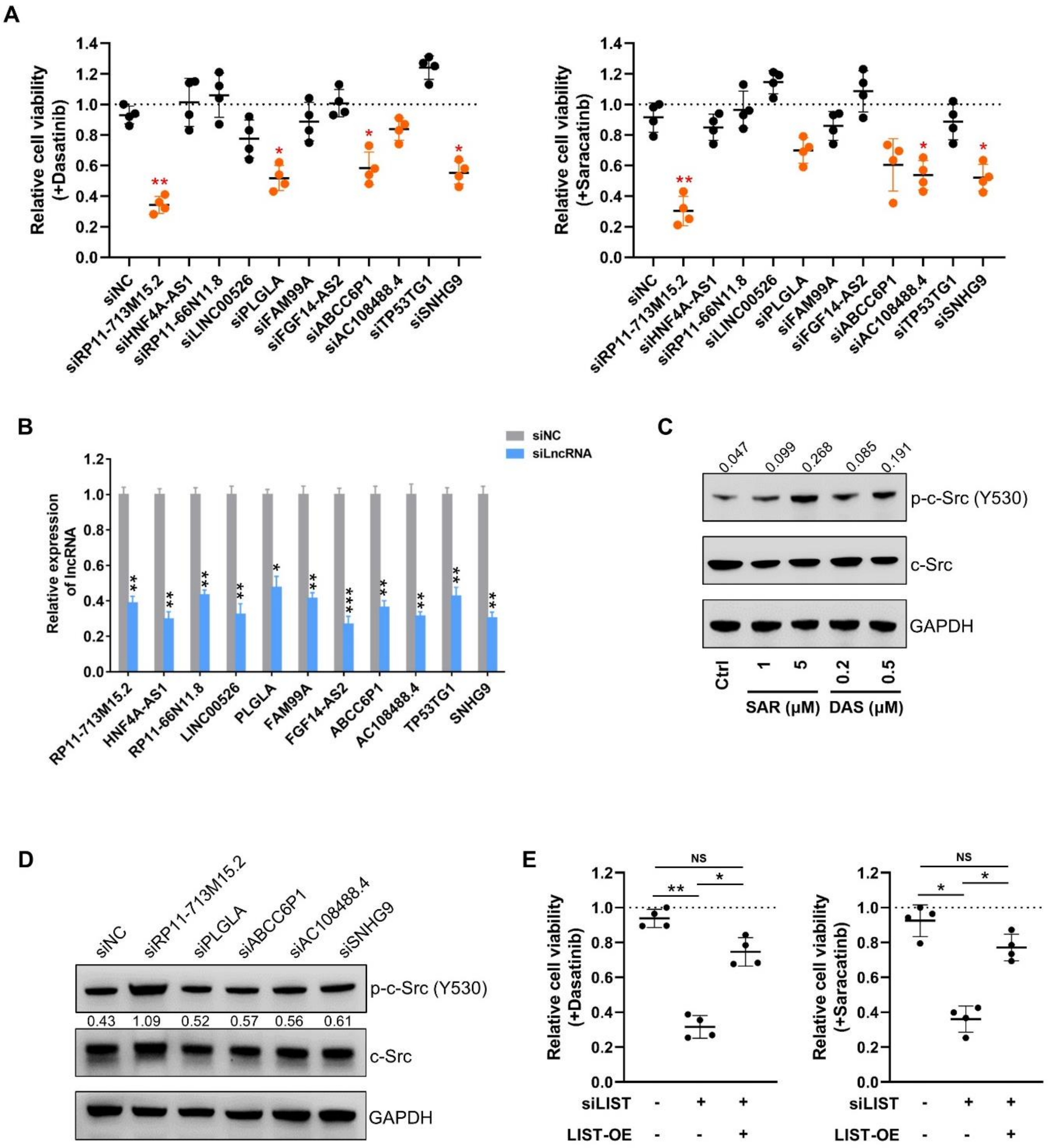
A
Relative cell viability (+Dasatinib)
Relative cell viability (+Saracatinb)
1.4
1.2
1.0
0.8
0.6
0.4
0.2
0.0
**
*
siNC
siRP11-713M15.2
siHNF4A-AS1
siRP11-66N11.8
siLINC00526
siPLGLA
siFAM99A
siFGF14-AS2
siABCC6P1
siAC108488.4
siTP53TG1
siSNHG9
B
siNC
siLncRNA
Relative expression of lncRNA
***
RP11-713M15.2
HNF4A-AS1
RP11-66N11.8
LINC00526
PLGLA
FAM99A
FGF14-AS2
ABCC6P1
AC108488.4
TP53TG1
SNHG9
C
0.047
0.099
0.268
0.085
0.191
p-c-Src (Y530)
c-Src
GAPDH
Ctrl
1
5
0.2
0.5
SAR (μM)
DAS (μM)
D
siNC
siRP11-713M15.2
siPLGLA
siABCC6P1
siAC108488.4
siSNHG9
p-c-Src (Y530)
0.43
1.09
0.52
0.57
0.56
0.61
c-Src
GAPDH
E
NS
siLIST
LIST-OE

Fig. S1.

***LIST* inhibited the phosphorylation level of c-Src.** (A). The 5637 cell activity was examined using the CellTiter-Glo Kit upon lncRNA knockdown after cells were treated with a c-Src inhibitor. Dasatinib (0.5 μM) and Saracatinib (5 μM) served as c-Src inhibitors. Error bars represent the SD of the four replicates (*P-value < 0.05, **P-value < 0.01), Student's t-test. (B). The relative expression levels of lncRNAs were measured by qPCR in 5637 cell line with corresponding siRNA-mediated knockdown. The error bars represent the SD of three replicates (*P-value < 0.05, **P-value < 0.01), Student's t-test. (C). The total protein and phosphorylation levels of c-Src were measured by western blotting in 5637 cells treated with c-Src inhibitors (dasatinib (DAS) and saracatinib (SAR)). The proteins were quantified by Image J software. The numbers represent the ratio of p-c-Src-Y530/c-Src. (D). The total protein and phosphorylation levels of c-Src were detected in gemcitabine-resistant bladder cancer cells (the 5637-GEM cell line) cells by western blotting upon lncRNAs knockdown. The proteins were quantified by Image J software. The numbers represent the ratio of p-c-Src-Y530/c-Src. (E) The effect of *LIST*-rescued expression on cell activity was examined using the CellTiter-Glo Kit in cells treated with a c-Src inhibitor. Dasatinib (0.5 μM) and Saracatinib (5 μM) served as c-Src inhibitors. Error bars represent the SD of the four replicates (NS represents not significant, *P-value < 0.05, **P-value < 0.01), Student's t-test.

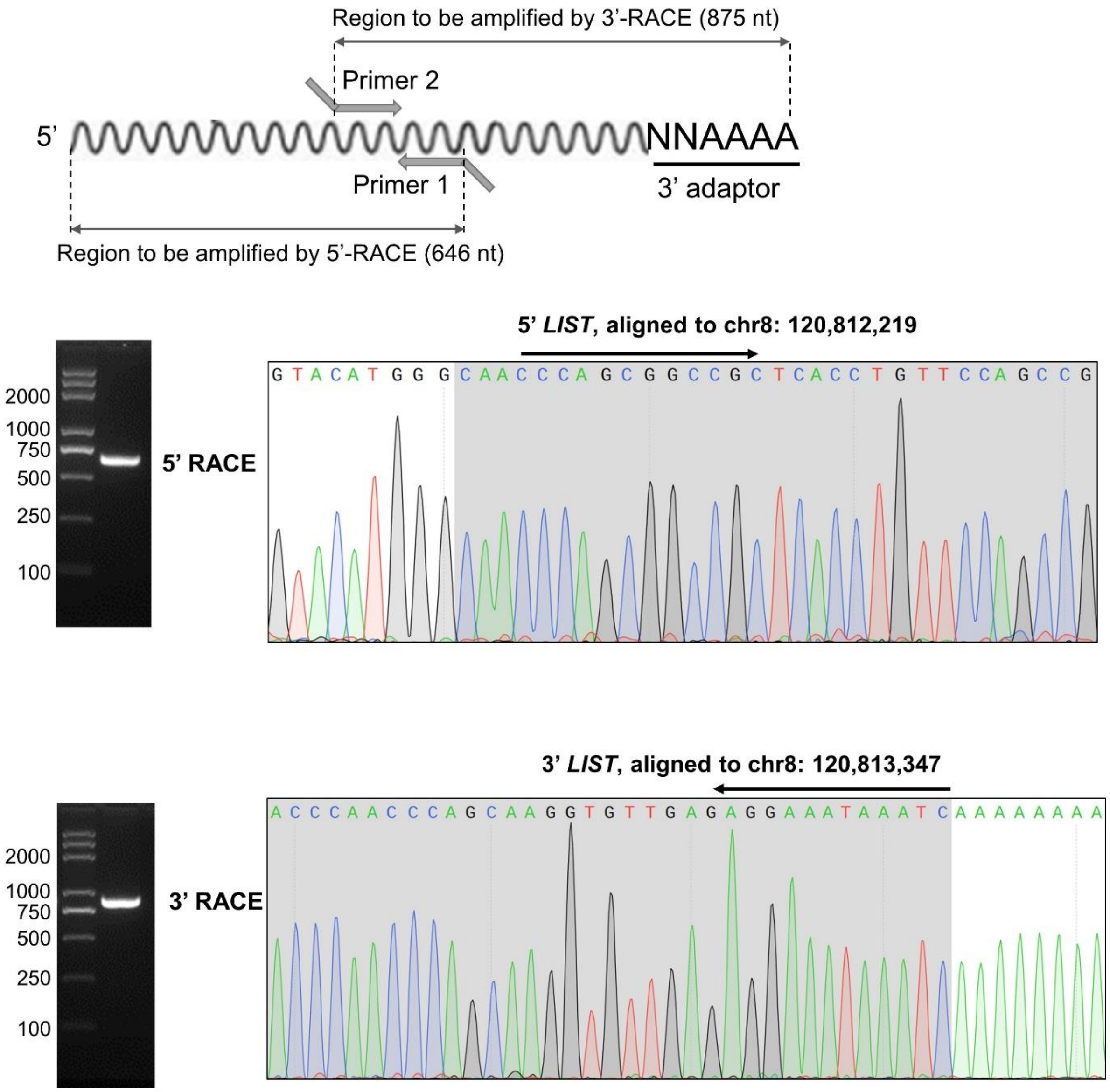

Fig. S2.

Confirmation of the *LIST* annotations on the genome. Top: Schematic representation of 5’- and 3’-RACE assays. Bottom: The *LIST* sequence was validated by agarose gel electrophoresis (left) and Sanger sequencing (right) of 5'-RACE and 3'-RACE PCR products in 5637 cells.

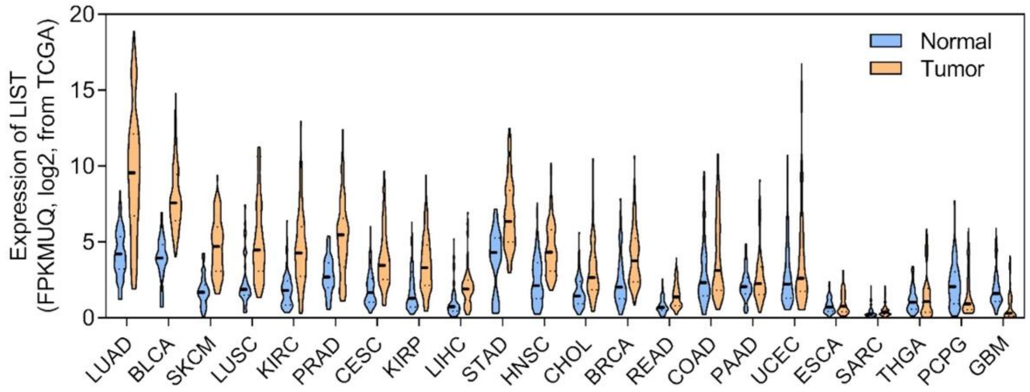

Fig. S3.

***LIST* is expressed in cancers.** RNA expression levels of *LIST* in TCGA tumors and corresponding normal tissues. Data were obtained from 22 cancer types in the TCGA database. Ranked from left to right according to differences in *LIST* expression.

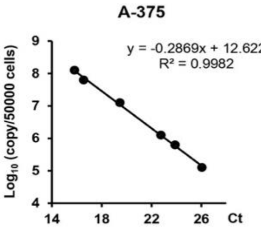

	Ct	copy/cell
LIST	22.15	78.55
HOTTIP	22.06	83.26
ACTIN	13.52	20819.14

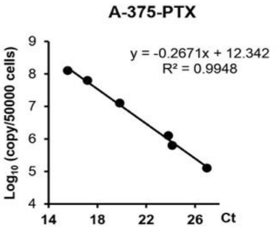

	Ct	copy/cell
LIST	20.81	230.44
HOTTIP	22.15	94.41
ACTIN	14.36	19958.81

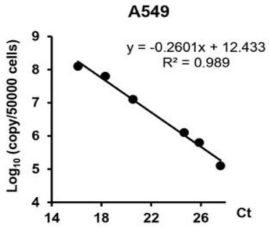

	Ct	copy/cell
LIST	22.19	101.93
HOTTIP	22.43	87.06
ACTIN	13.97	22523.44

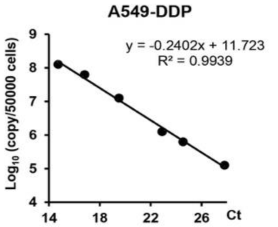

	Ct	copy/cell
LIST	20.07	297.8
HOTTIP	22.59	61.87
ACTIN	13.12	22697.75

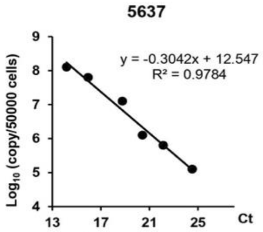

	Ct	copy/cell
LIST	20.11	89.62
HOTTIP	21.98	24.19
ACTIN	12.47	18901.87

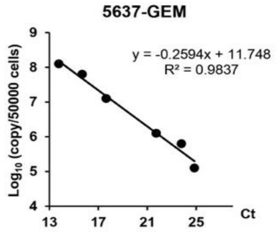

	Ct	copy/cell
LIST	18.23	348.35
HOTTIP	21.57	47.38
ACTIN	11.39	20654.55

Fig. S4.

The RNA copy numbers of *LIST* were measured. The RNA copy numbers of *LIST* were calculated in six cancer cells. For each cancer cell line, serial dilution standards (RNA spike-in) were added to the RNA samples, and a standard curve was generated by qPCR. Top: the standard curve of corresponding cancer cells, where the X-axis represents the Ct values from qPCR and the Y-axis represents the copy numbers (log10) of the standard added to the lysate from 50000 cells. Bottom: The average copy number of *LIST* (other transcripts as references) per cell was calculated by fitting Ct values to the standard curve.

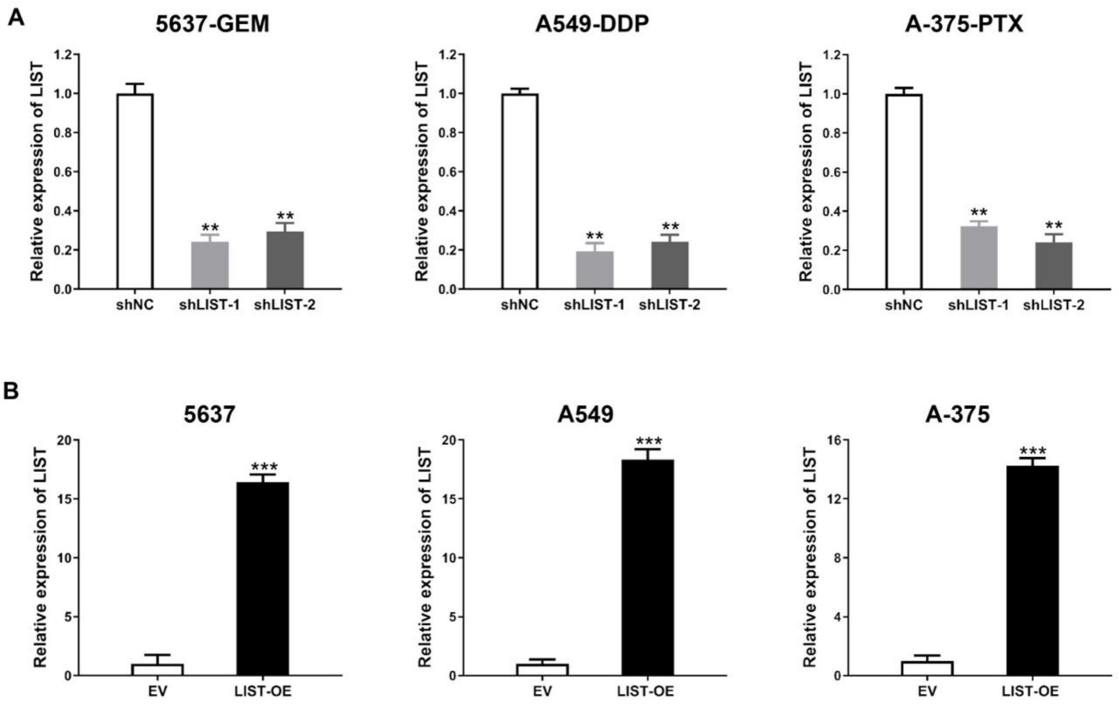

Fig. S5.

Relative expression levels of *LIST* in various cancer cells upon *LIST* knockdown or overexpression. (A). Relative expression levels of *LIST*, measured by qPCR, in various cancer cells with shRNA-mediated knockdown. The error bars represent the SD of three replicates. (B). Relative expression levels of *LIST*, measured by qPCR, in various cancer cells stably transfected with *LIST* overexpression. The error bars represent the SD of three replicates (**P-value < 0.01, ***P-value < 0.001), Student's t-test.

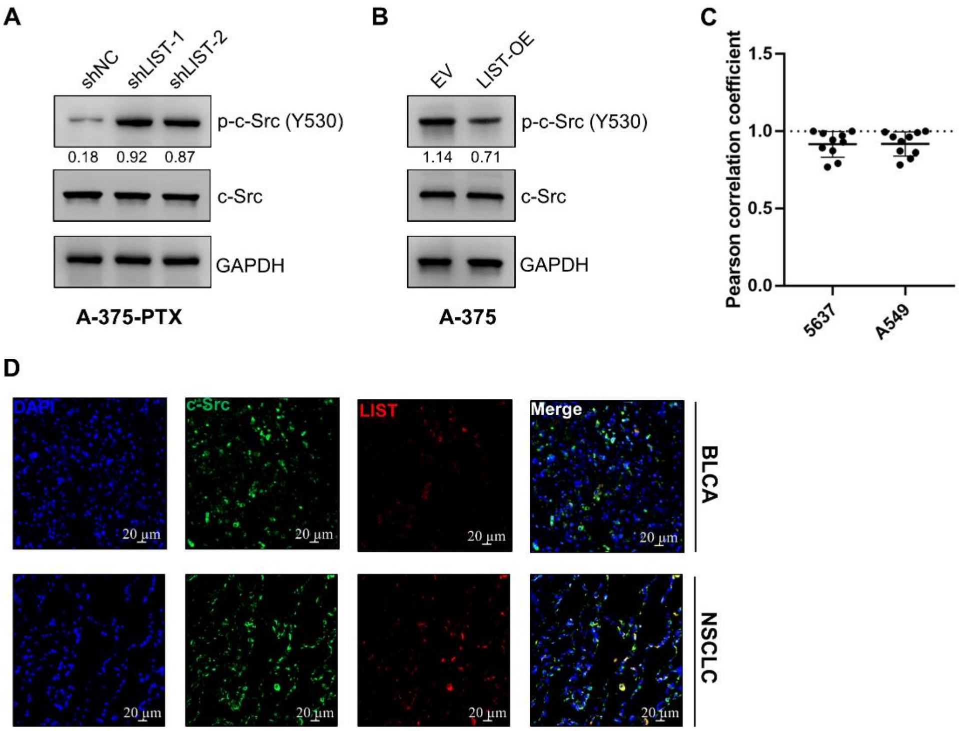

Fig. S6.

***LIST* binds to and inhibits the phosphorylation level of c-Src (Y530).** (A). The total protein and phosphorylation levels of c-Src were detected in paclitaxel-resistant melanoma cells by western blotting upon *LIST* knockdown. The proteins were quantified by Image J software. The numbers represent the ratio of p-c-Src-Y530/c-Src. (B). The total protein and phosphorylation levels of c-Src were detected in chemosensitive melanoma cells by western blotting upon *LIST* overexpression. (C). Supplementary to Fig. 1F. The degrees of co-localization between *LIST* and c-Src in cell lines 5637 and A549 were quantified by the Pearson's correlation coefficients (PCC). The maximal value of PCC (1.0) indicates perfect co-localization between two fluorescence signals in a cell, whereas PCC=0 indicates no co-localization. See Methods for the procedure of calculating the PCC values. (D). Co-localization immunofluorescence staining of *LIST* (red) and c-Src (green) in BLCA and NSCLC cancer tissues. Nuclei were stained with DAPI (blue). Scale bar: 20µm.

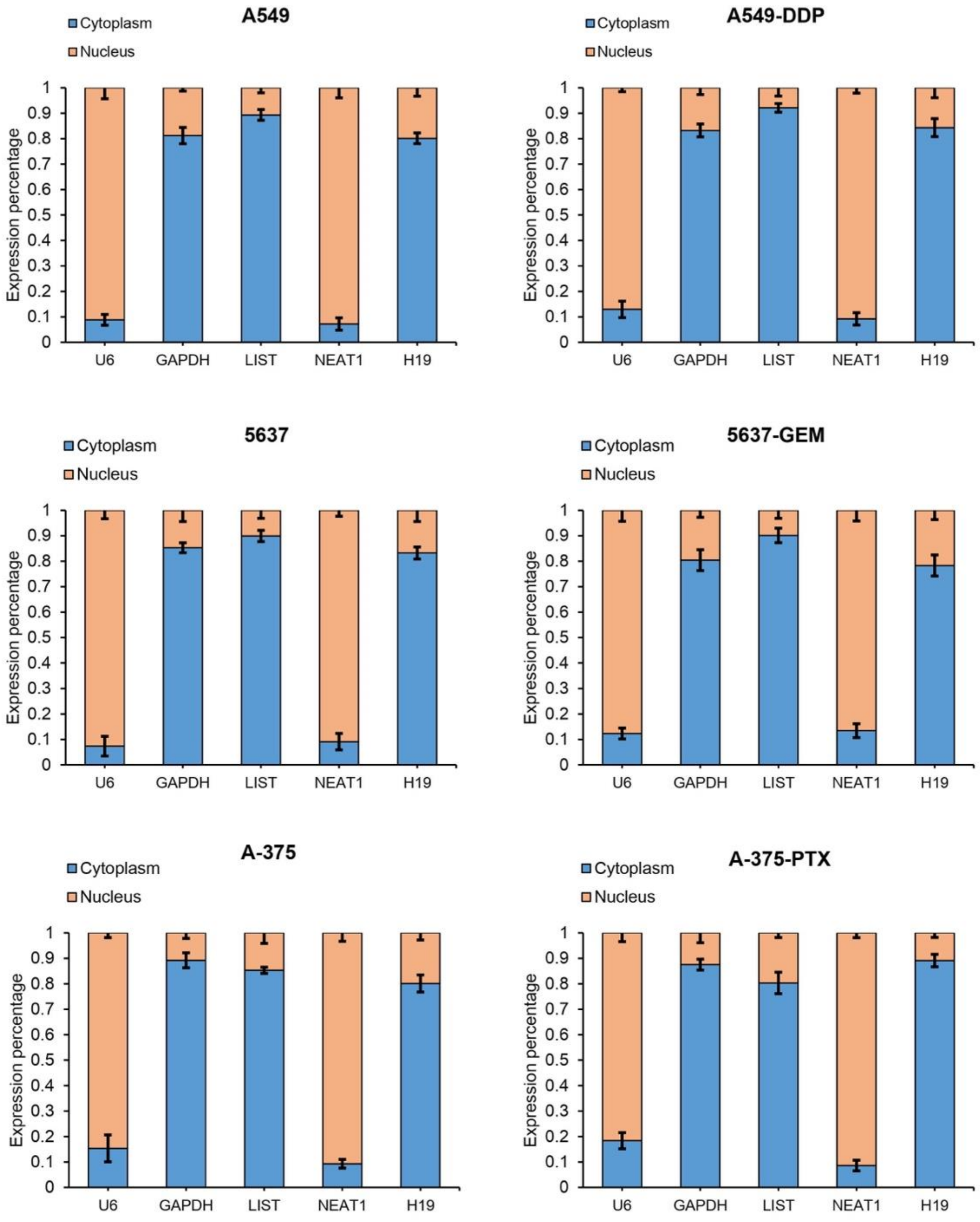

Fig. S7.

Subcellular localization of *LIST* in cancer cells. The nuclear and cytosolic fractions of *LIST* expression were detected using RT-qPCR. GAPDH and H19 served as cytosolic markers, while NEAT1 and U6 served as nuclear markers.

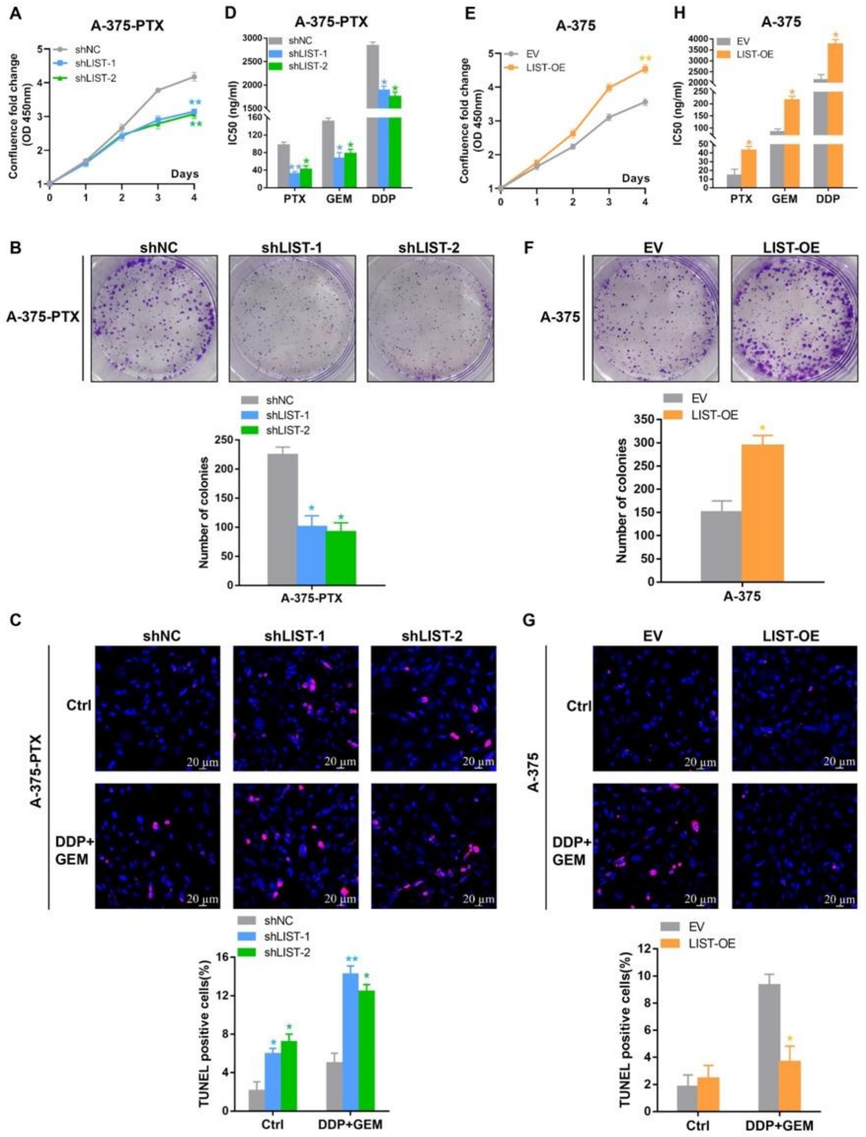

Fig. S8.

The function of *LIST* in melanoma proliferation, apoptosis, and chemoresistance. (A-B). Paclitaxel-resistant A375 cells (A-375-PTX) were examined for cell proliferation using the CCK-8 assay (A) and colony formation assay (B) after *LIST* knockdown. (C). The TUNEL assay was used to identify apoptosis (red) in A-375-PTX cells with *LIST* knockdown. DDP (cisplatin) 5 μM; gemcitabine (GEM) 5 μM. (D). The CellTiter-Glo Kit was used to determine the IC50 value of the drug after *LIST* knockdown. (E-F). The CCK-8 assay (E) and colony formation assay (F) were used to assess the proliferation of A-375 cells stably transfected with *LIST* overexpression. (G). Apoptotic cells (red) were stained using the TUNEL assay in A-375 cells following *LIST* overexpression. DDP (cisplatin) 5 μM; gemcitabine (GEM) 5 μM. (H). Drug IC50 value of A-375 cells stably transfected with *LIST* overexpression was examined using the CellTiter-Glo Kit. (A-H). Error bars show the standard deviation (SD) of three replicates (*P-value < 0.05, **P-value < 0.01), Student's t-test.

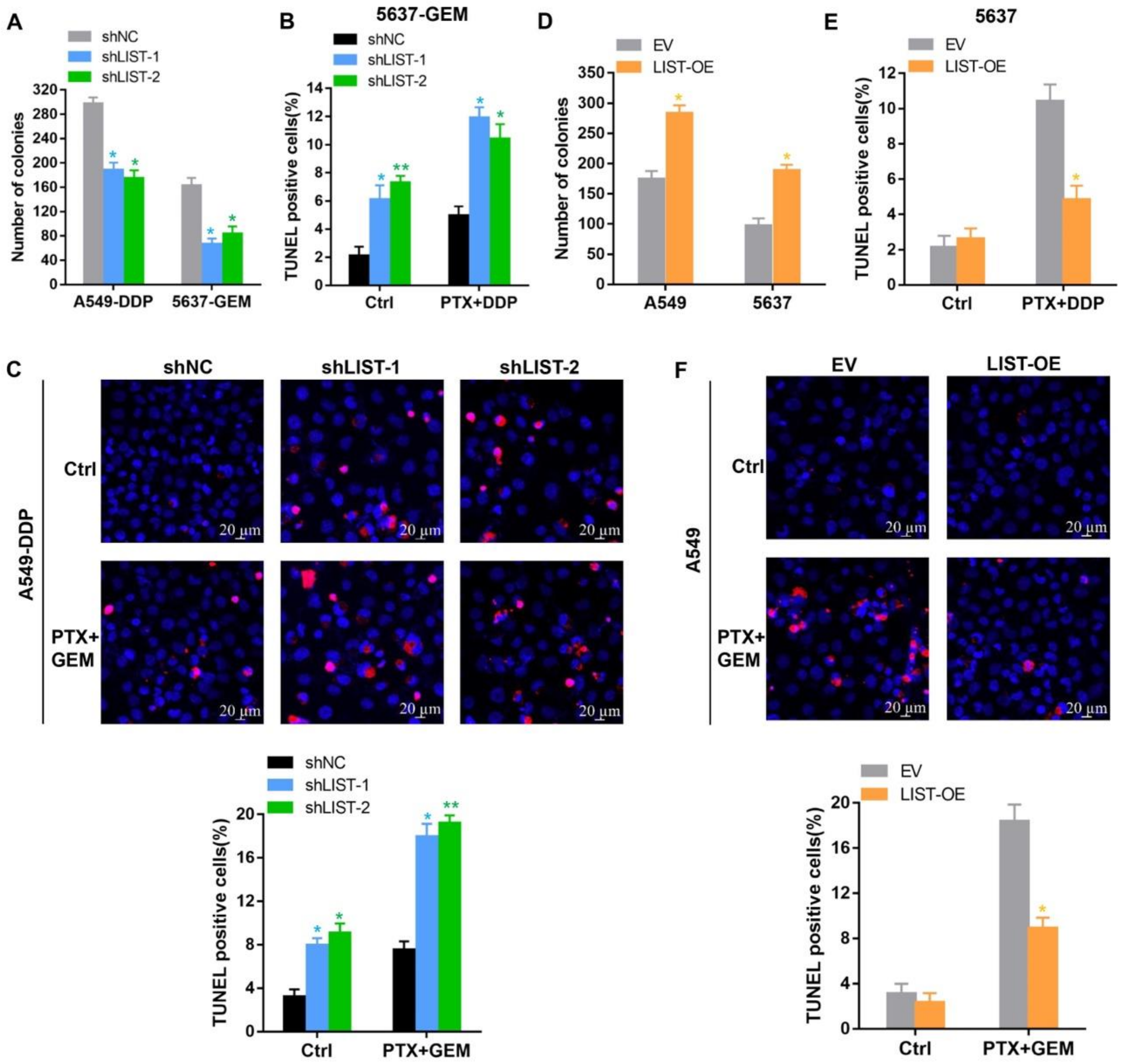

Fig. S9.

The function of *LIST* in cell proliferation and apoptosis. (A). Supplementary to Fig. 3B, it shows the statistical examination of colony formation in 5637-GEM and A549-DDP cells. (B). Supplementary to Fig. 3C, apoptotic cells of 5637-GEM were analyzed statistically. (C). The TUNEL assay was performed to detect apoptosis (red) in A549-DDP cells with *LIST* knockdown. PTX (paclitaxel), 0.05μM; gemcitabine (GEM), 5 μM. (D). Supplementary to Fig. 3F, a statistical examination of colony formation in 5637 and A549 cells was performed. (E). Supplementary to Fig. 3G, the apoptosis of 5637 cells was statistically analyzed. (F). Apoptotic cells (red) of A549 cells stably transfected with *LIST* overexpression were stained using the TUNEL assay. PTX (paclitaxel), 0.05 μM; gemcitabine (GEM), 5 μM. (A-F). Error bars show the standard deviation (SD) of three replicates (*P-value < 0.05, **P-value < 0.01), Student's t-test.

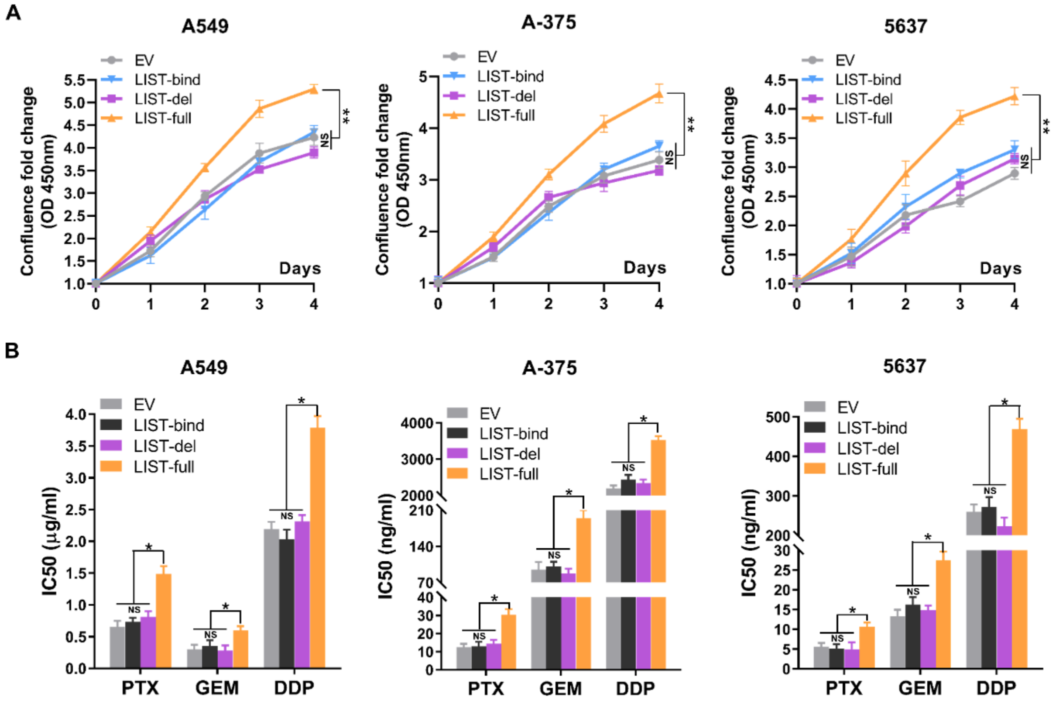

Fig. S10.

Function of *LIST* truncates in cancer cell proliferation and chemoresistance. (A). Cell proliferation curves of cancer cells upon *LIST*-binding (containing only two c-Src-binding fragments, 1 and 6), *LIST*-del (lacking the two c-Src-binding fragments, 1 and 6), or *LIST*-full (full length) overexpression were analyzed using CCK-8. (B). The drug IC50 value was examined using the CellTiter-Glo Kit upon *LIST*-binding (containing only two c-Src-binding fragments (1 and 6)), *LIST*-del (lacking the two c-Src-binding fragments (1 and 6)), or *LIST*-full (full-length) overexpression in various cancer cells. (A-B). Error bars show the standard deviation (SD) of three replicates (NS represents not significant, *P-value < 0.05, **P-value < 0.01), Student's t-test.

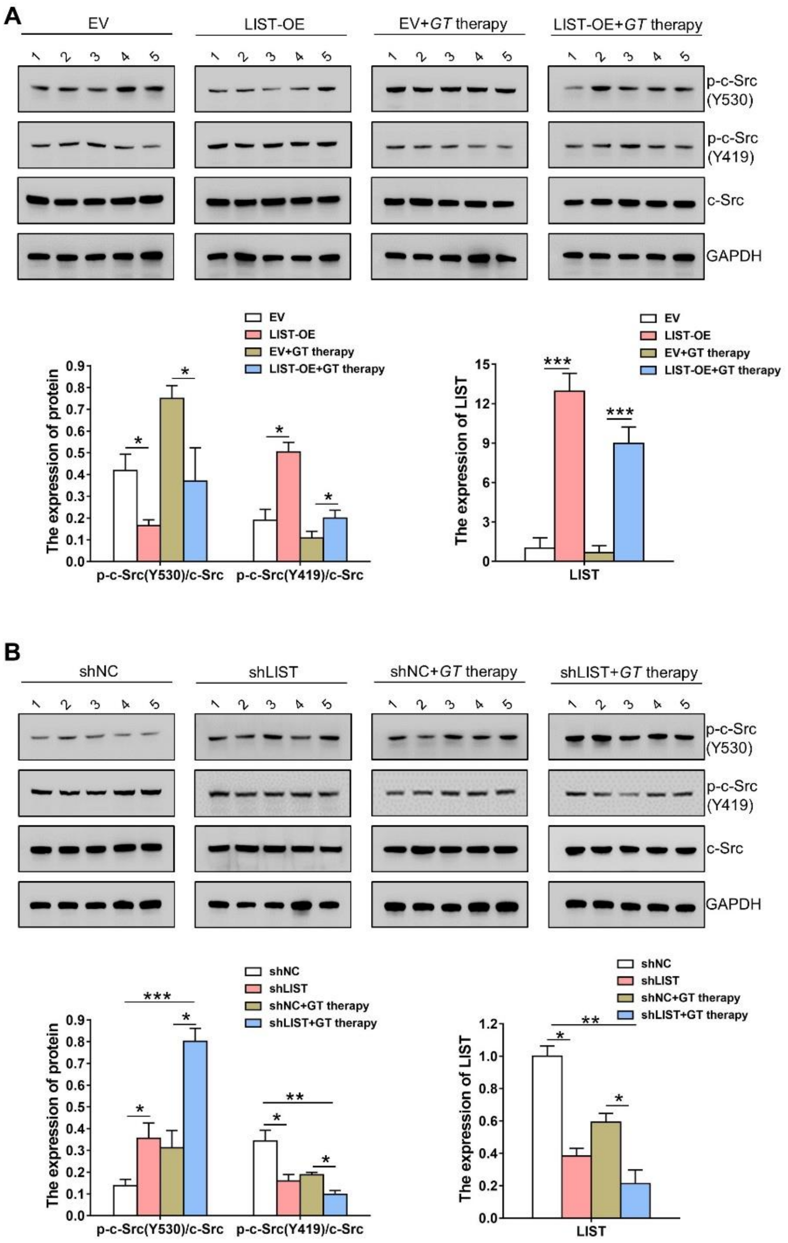

Fig. S11.

The expression levels of c-Src and *LIST* in xenograft tumor models. (A). Supplementary to Fig. 4A. The expression levels of c-Src(Y530, Y419) and *LIST* in xenograft tumor models from A549 cells were examined via western blotting or qRT-PCR. (B). Supplementary to Fig. 4B. The expression levels of c-Src(Y530, Y419) and *LIST* in xenograft tumor models from A549-DDP cells were examined via western blotting or qRT-PCR. The proteins were quantified by Image J software. The ratio of p-c-Src-Y530/c-Src or p-c-Src-Y419/c-Src was analyzed using GraphPad Prism 8. (A-B). Error bars show the standard deviation (SD) of five replicates (*P-value < 0.05, **P-value < 0.01, ***P-value < 0.001), Student's t-test.

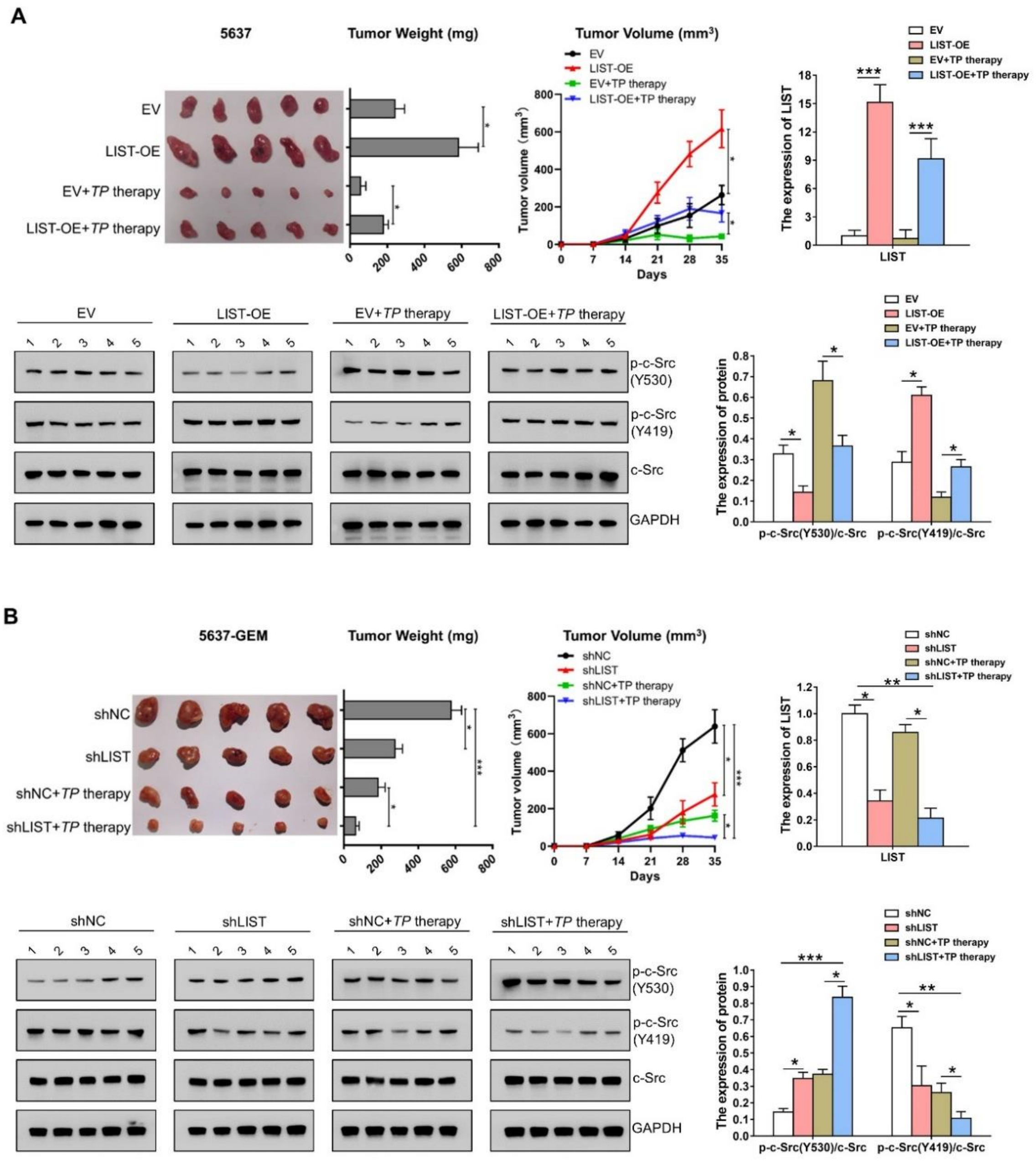

Fig. S12.

The function of *LIST* in xenograft tumor models. (A-B). The impact of *LIST* overexpression (A) and knockdown (B) on tumor growth and chemoresistance *in vivo* was investigated using xenograft tumor models. The tumor images, weights, and growth trajectories are displayed. Cisplatin and paclitaxel are used in combination therapy, known as *TP* therapy. The expression levels of c-Src(Y530, Y419) and *LIST* in xenograft tumor models were examined via western blotting or qRT-PCR. The proteins were quantified by Image J software. The ratio of p-c-Src-Y530/c-Src or p-c-Src-Y419/c-Src was analyzed using GraphPad Prism 8. Error bars represent the mean ± SD of the tumors. (A-B). Error bars show the standard deviation (SD) of five replicates (*P-value < 0.05, **P-value < 0.01, ***P-value < 0.001), Student's t-test.

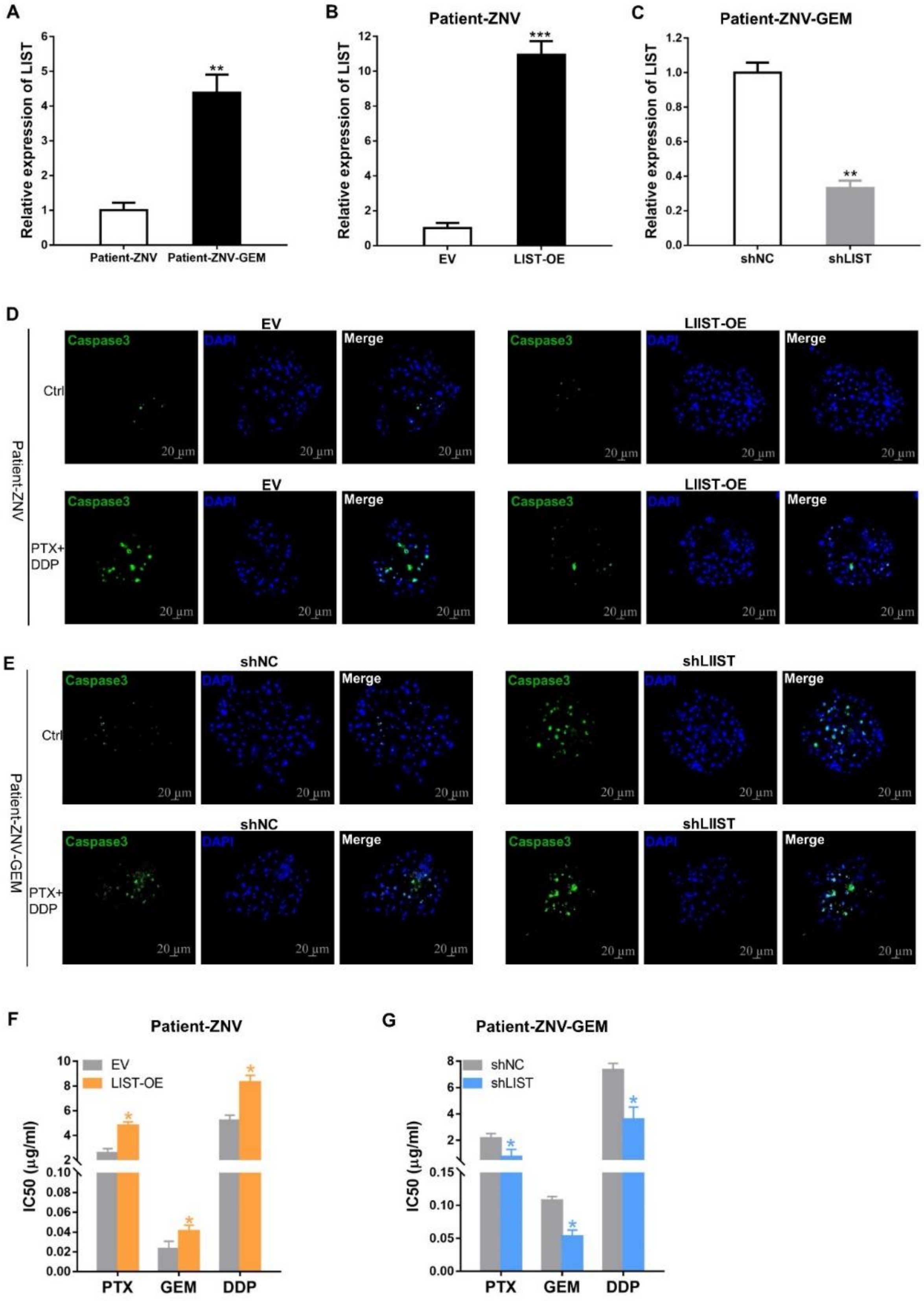
A
Relative expression of LIST
Patient-ZNV
Patient-ZNV-GEM
B
Patient-ZNV
EV
LIST-OE
C
Patient-ZNV-GEM
shNC
shLIST
D
EV
LIIST-OE
Caspase3
DAPI
Merge
Ctrl
PTX+
DDP
Patient-ZNV
E
shNC
shLIIST
Patient-ZNV-GEM
F
Patient-ZNV
IC50 (μg/ml)
PTX
GEM
DDP
G
Patient-ZNV-GEM

Fig. S13.

The function of *LIST* in organoid model of bladder carcinoma. (A). Relative expression levels of *LIST*, measured by qPCR, in bladder carcinoma organoid and gemcitabine-resistant organoid. (B). Relative expression levels of *LIST*, measured by qPCR, in bladder carcinoma organoid stably transfected with *LIST* overexpression. The error bars represent the SD of three replicates. (C). Relative expression levels of *LIST*, measured by qPCR, in gemcitabine-resistant organoid with shRNA-mediated knockdown. The error bars represent the SD of three replicates. (D). Supplementary to Fig. 4E, apoptotic cells (green) were stained using Caspase 3/7 Activity Apoptosis Assay Kit upon *LIST* overexpression. PTX (paclitaxel), 0.05μM; DDP (cisplatin), 20μM. (E). Supplementary to Fig. 4F, apoptotic cells (green) were stained using Caspase 3/7 Activity Apoptosis Assay Kit upon *LIST* knockdown. PTX (paclitaxel), 0.05μM; DDP (cisplatin), 20μM. (F-G). Drug IC50 values of organoids stably transfected with *LIST* overexpression or knockdown were examined using the CellTiter-Glo Kit. (A-G). The error bars represent the SD of three replicates (*P-value < 0.05, **P-value < 0.01, ***P-value < 0.001), Student's t-test.

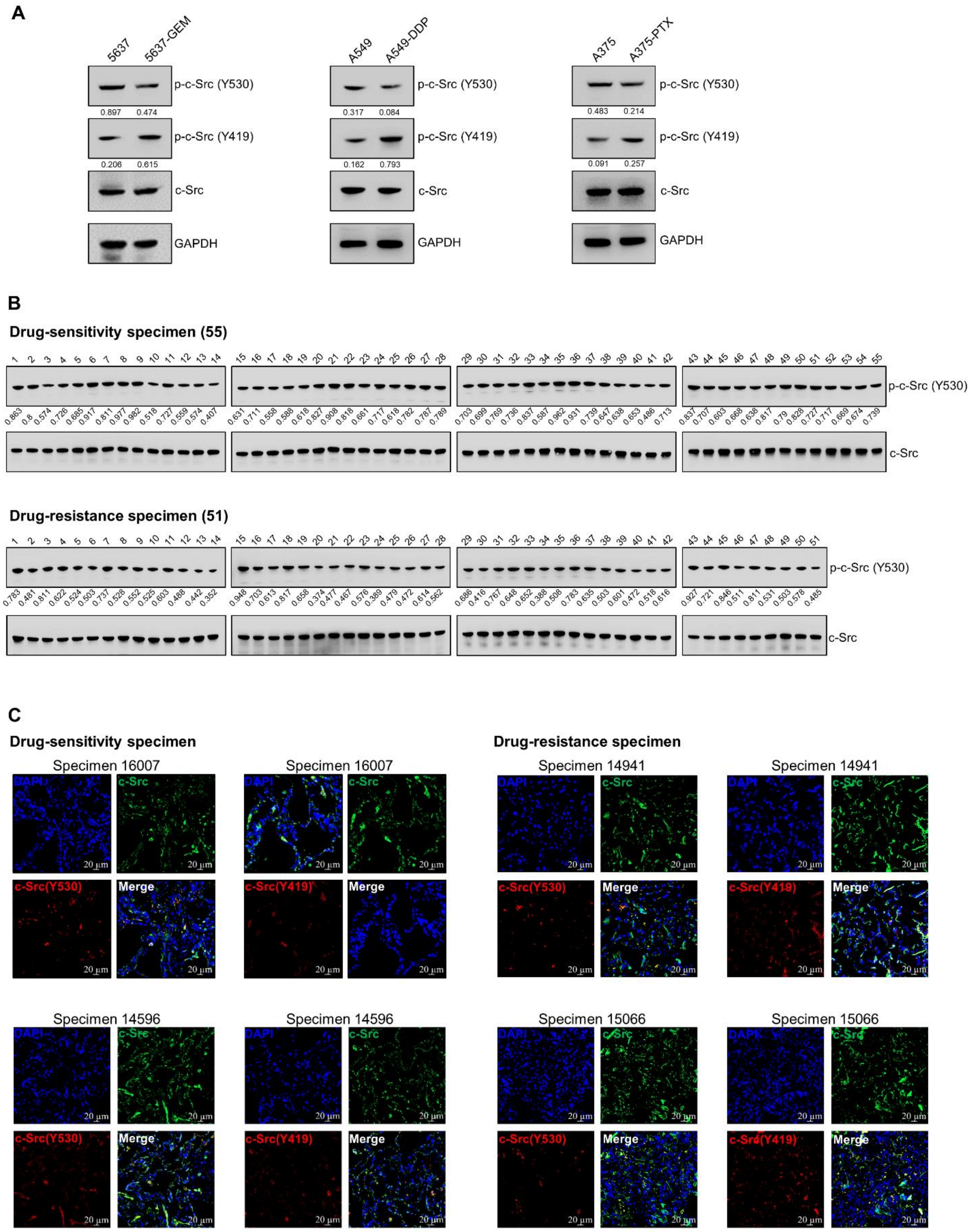
A
5637
5637-GEM
A549
A549-DDP
A375
A375-PTX
p-c-Src (Y530)
0.897 0.474
0.317 0.084
0.483 0.214
p-c-Src (Y419)
0.206 0.615
0.162 0.793
0.091 0.257
c-Src
GAPDH
B
Drug-sensitivity specimen (55)
Drug-resistance specimen (51)
p-c-Src (Y530)
c-Src
C
Drug-sensitivity specimen
Drug-resistance specimen
Specimen 16007
Specimen 14941
Specimen 14596
Specimen 15066
DAPI
c-Src
c-Src(Y530)
c-Src(Y419)
Merge
20 μm

Fig. S14.

The expression level of c-Src (Y530, Y419) in drug-sensitivity and drug-resistance groups. (A). The expression level of c-Src (Y530, Y419) in the chemoresistant cell lines and chemosensitive cells was detected via western blotting. The proteins were quantified by Image J software. The numbers represent the ratio of p-c-Src-Y530/c-Src or p-c-Src-Y419/c-Src. (B). Supplementary to Fig. 5C. The expression level of c-Src (Y530) was examined in the drug-sensitivity and drug-resistance groups. via western blotting. The proteins were quantified by Image J software. The numbers represent the ratio of p-c-Src-Y530/c-Src. (B). Co-localization immunofluorescence staining of c-Src (green) and c-Src (Y530, Y419) (red) in NSCLC cancer tissues. Nuclei were stained with DAPI (blue). Scale bar: 20μm..

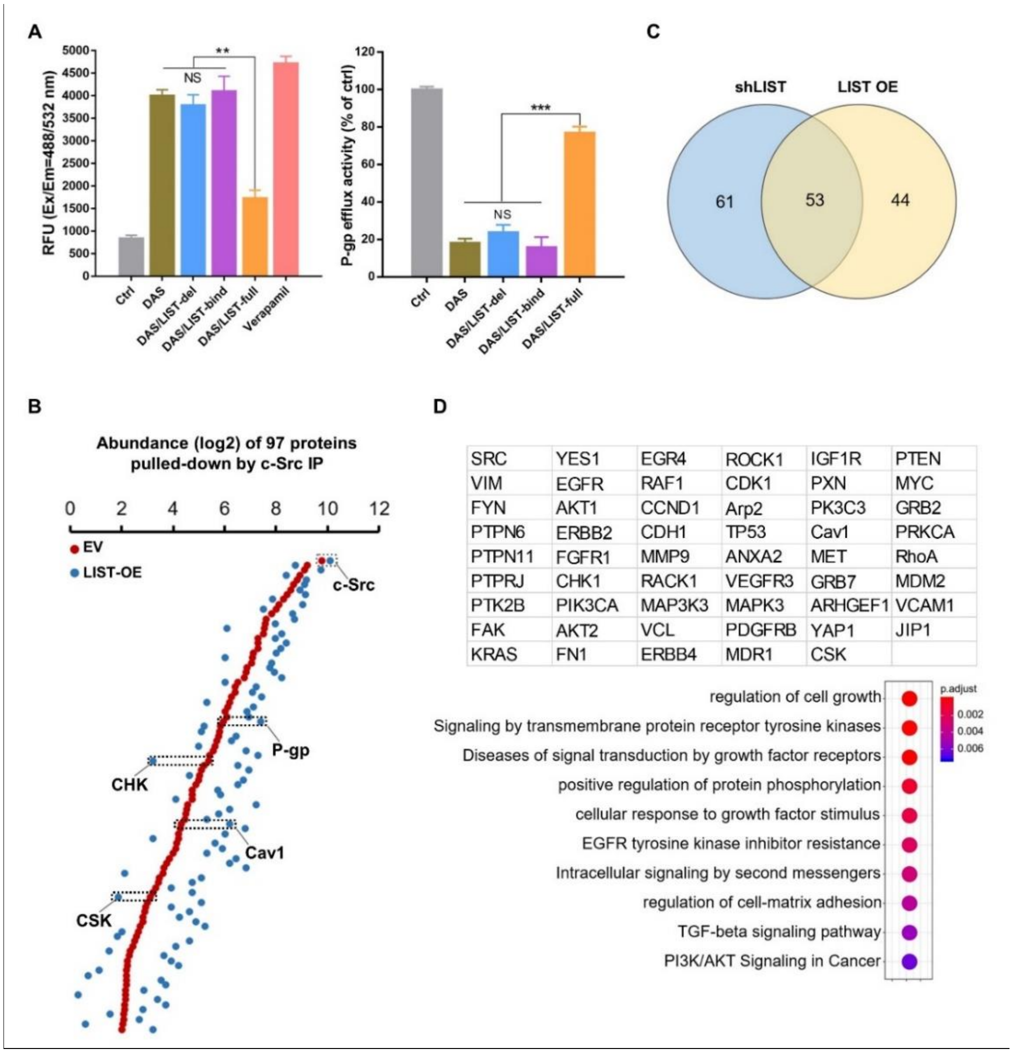

Fig. S15.

***LIST* regulated c-Src molecular functions.** (A). Left: the intracellular accumulation of fluorogenic P-gp substrate hydrolysis product was measured in different condition treated 5637-GEM cells via the fluorescence intensity (Ex/Em = 488/532 nm), Verapamil (P-gp Inhibitor) served as positive control; Right: the P-gp efflux activity was calculated by the following equation: % Activity =100-[(Fr-Fc)/(Fm-Fc)] ×100. Fc is the fluorescence intensity of the no inhibition control condition (control group), Fm is the the fluorescence intensity of verapamil and Fr is the fluorescence intensity of the cells under different conditions. The error bars represent the standard deviation (SD) of three replicates (NS represents not significant, **P-value < 0.01, ***P-value < 0.001), Student's t-test. (B). Mass spectrometry study of the proteins downregulated in *LIST*-overexpressing or control cells after c-Src immunoprecipitation. The most prevalent protein, c-Src, which was consistent between the groups, as predicted, served as a positive control. (C). Overlap between the two sets of proteins pulled out by c-Src under the conditions of *LIST* knockdown or overexpression. (D). Functional enrichment of GO and KEGG in overlapping c-Src-binding proteins.

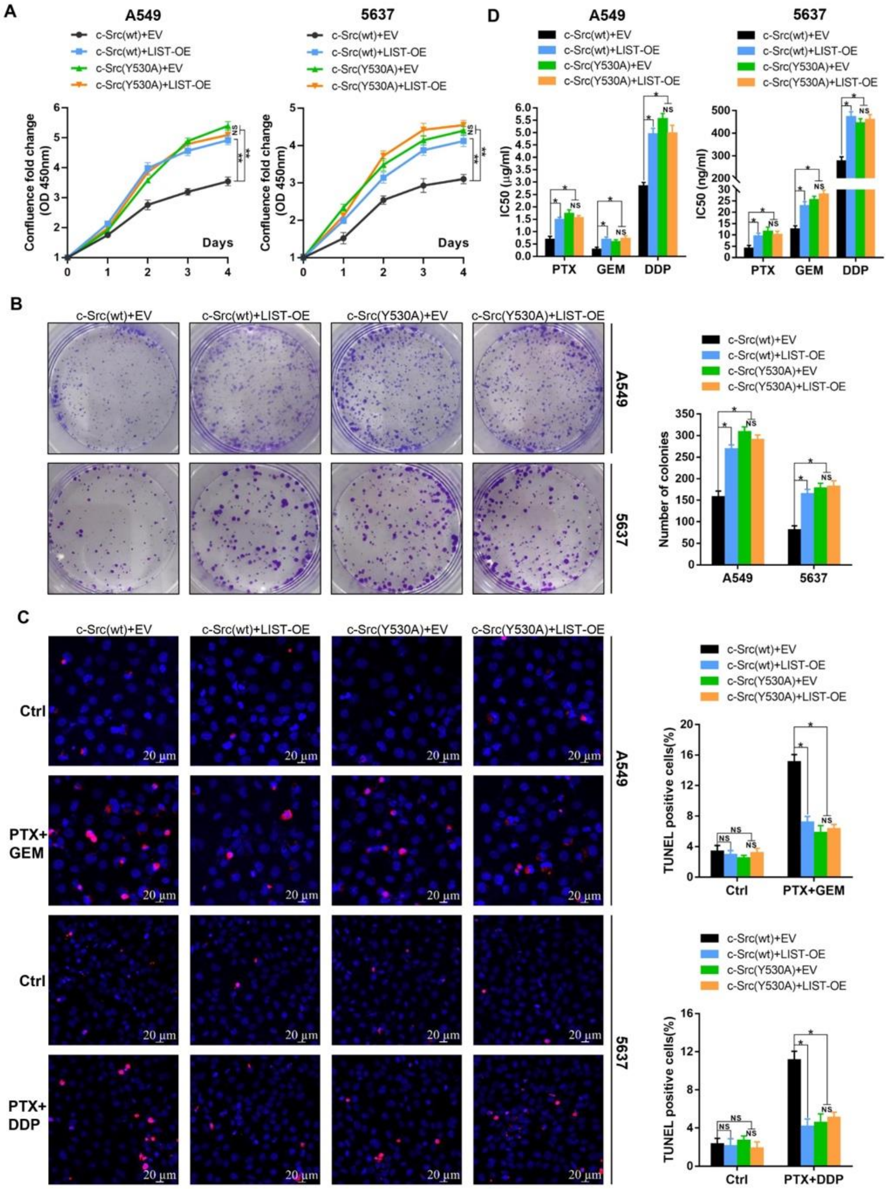

Fig. S16.

The biological functions of c-Src were regulated by *LIST*. (A-D). In c-Src$^{-/-}$ cells created using the CRISPR/CAS9 system, c-Src wild-type (c-Src(wt)) or c-Src mutant (c-Src(Y530A), where Y530 mimics dephosphorylation, Y-A) was reintroduced. Upon *LIST* overexpression, the capacity of these cells to proliferate was assessed using the CCK-8 assay (A) and colony formation assay (B). (C) The TUNEL assay was used to examine apoptotic cells (red) in response to *LIST* overexpression. (D) The CellTiter-Glo Kit was used to determine the IC50 value of the drug in these cells following *LIST* overexpression. A549 and 5637 cells were used for each experiment. DDP (cisplatin), 5 μM; gemcitabine (GEM), 5 μM; paclitaxel (PTX), 0.05μM. Error bars show the SD of the three replicates (NS represents not significant, *P-value < 0.05, **P-value < 0.01), Student's t-test.

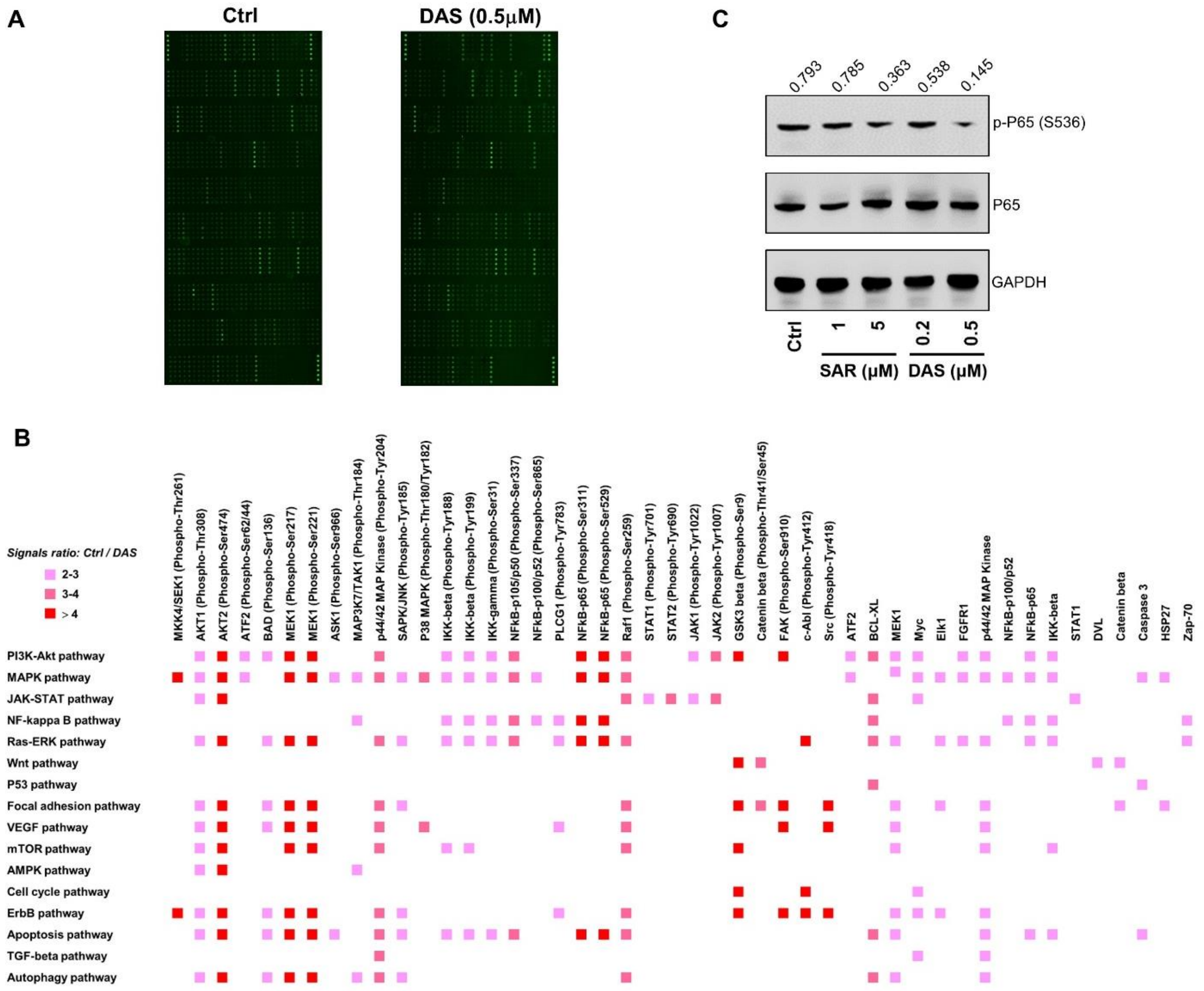

Fig. S17.

Several key signaling pathways were regulated by c-Src. (A-B). The expression and activation of key signaling molecules were examined with or without c-Src inhibitor (DAS). Phospho-array for proteins **(**A) and proteins with Ctrl/DAS ratios greater than 2 **(**B) are shown; the signal values of these proteins were shown in Supplementary Table. S1. **(**C). The total protein and phosphorylation levels of P65 were measured by western blotting in 5637-GEM cells treated with c-Src inhibitors (dasatinib (DAS) and saracatinib (SAR)). The proteins were quantified by Image J software. The numbers represent the ratio of p-P65-S536/P65.

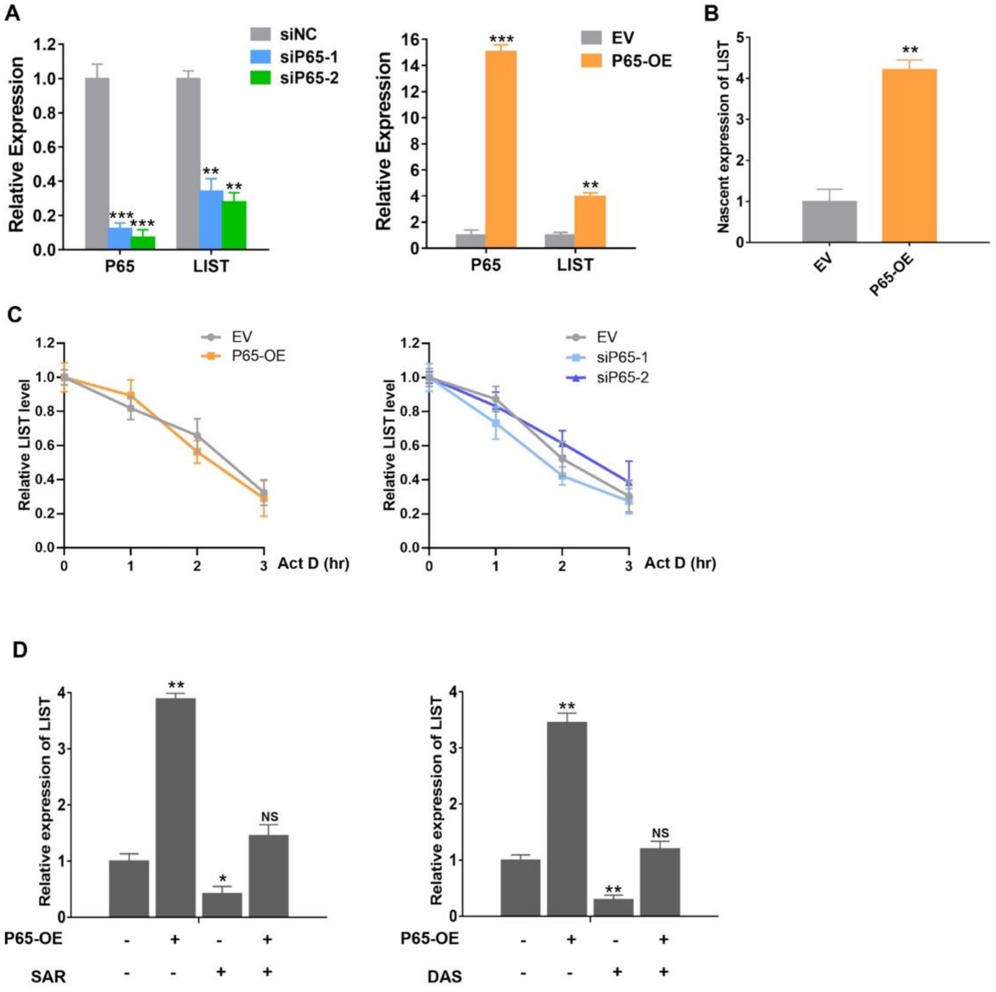

Fig. S18.

The c-Src/P65 axis regulated the expression of *LIST*. (A). The relative expression levels of *LIST* were determined by RT-qPCR in 5637-GEM cells with P65 knockdown and in 5637 cells with P65 overexpression. (B). By capturing nascent RNA, the relative expression levels of the nascent *LIST* transcript were determined in 5637 cells overexpressing P65. (C). The rate of RNA degradation was investigated in cancer cells treated with actinomycin D (5μM), which inhibits general transcription. (D). Changes in the expression of *LIST* were examined in 5637 cells overexpressing p65 after treatment with various conditions. Dasatinib (DAS) and Saracatinib (SAR) are c-Src inhibitors. (A-D). The error bars represent the SD of three replicates (NS represents not significant, *P-value < 0.05, **P-value < 0.01, ***P-value < 0.001), Student's t-test.

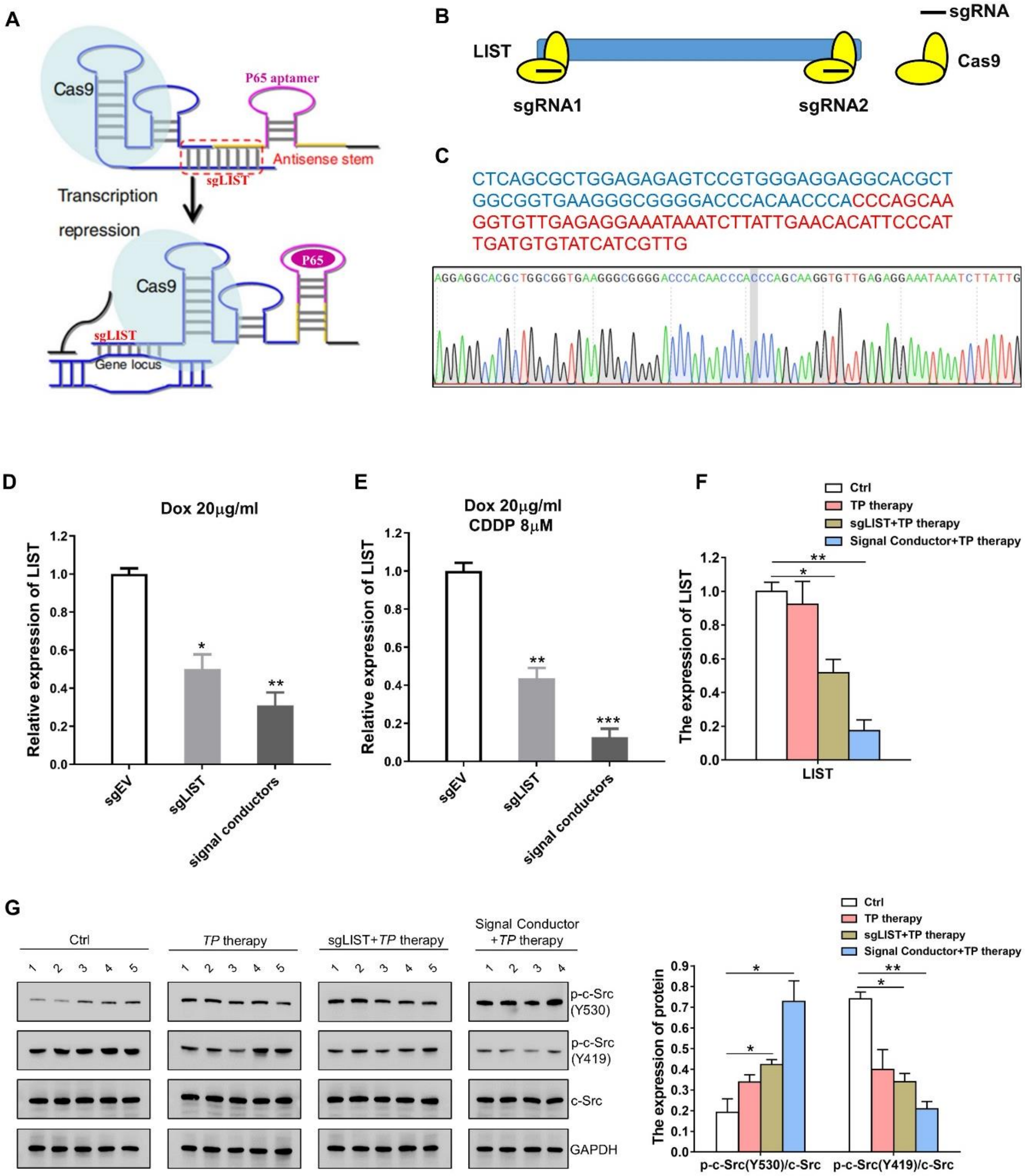

Fig. S19.

The "signal conductor" controls the expression of *LIST*. (A). Schematic diagram of the CRISPR/Cas9-based 'signal conductor': The regulatory element was first constructed by adding the p65 aptamer sequence to the 3' end of the gRNA sequence of LIST. (i) When the NF-κB pathway was not triggered, the element was unable to bind to the P65 protein, at which point the element was in a quiescent state because a portion of the P65 aptamer sequence was complementary to the gRNA sequence. (ii) When the NF-κB pathway is activated, the aptamer binds to the P65 protein in a competitive manner, and the RNA conformation changes in the

element to release the gRNA sequence, which guides the Cas9 protein to cleave the *LIST* gene to achieve gene knockout; simultaneous aptamer binding to P65 prevents P65 from binding to the promoter of *LIST*, inhibiting its transcriptional activation. Finally, dual regulation targeting *LIST* is achieved. (B). Strategy for *LIST* knockdown with the Tet-on CRISPR/Cas9 system. (C). Sanger sequencing results demonstrated *LIST* knockout. Removal of the *LIST* segment between the two sgRNA targeting sites is indicated by the sequences in blue and red, which correspond to the sequences upstream of sgRNA1 and downstream of sgRNA2, respectively. (D-E). The knockout effect of *LIST* was measured on the "Tet-on CRISPR/Cas9 system" or "signal conductors" with or without cisplatin treatment. The error bars represent the standard deviation (SD) of three replicates (*P-value < 0.05, **P-value < 0.01, ***P-value < 0.001), Student's t-test. (F). The knockout effect of *LIST* was measured by qRT-PCR in xenograft tumors shown in Fig. 8H. (G). Supplementary to Fig. 8H. The expression levels of c-Src (Y530, Y419) in xenograft tumor models from 5637-GEM cells were examined via western blotting. The proteins were quantified by Image J software. The ratio of p-c-Src-Y530/c-Src or p-c-Src-Y419/c-Src was analyzed using GraphPad Prism 8. (F-G). Error bars represent the mean ± SD of the tumors (*P-value < 0.05, **P-value < 0.01), Student's t-test.

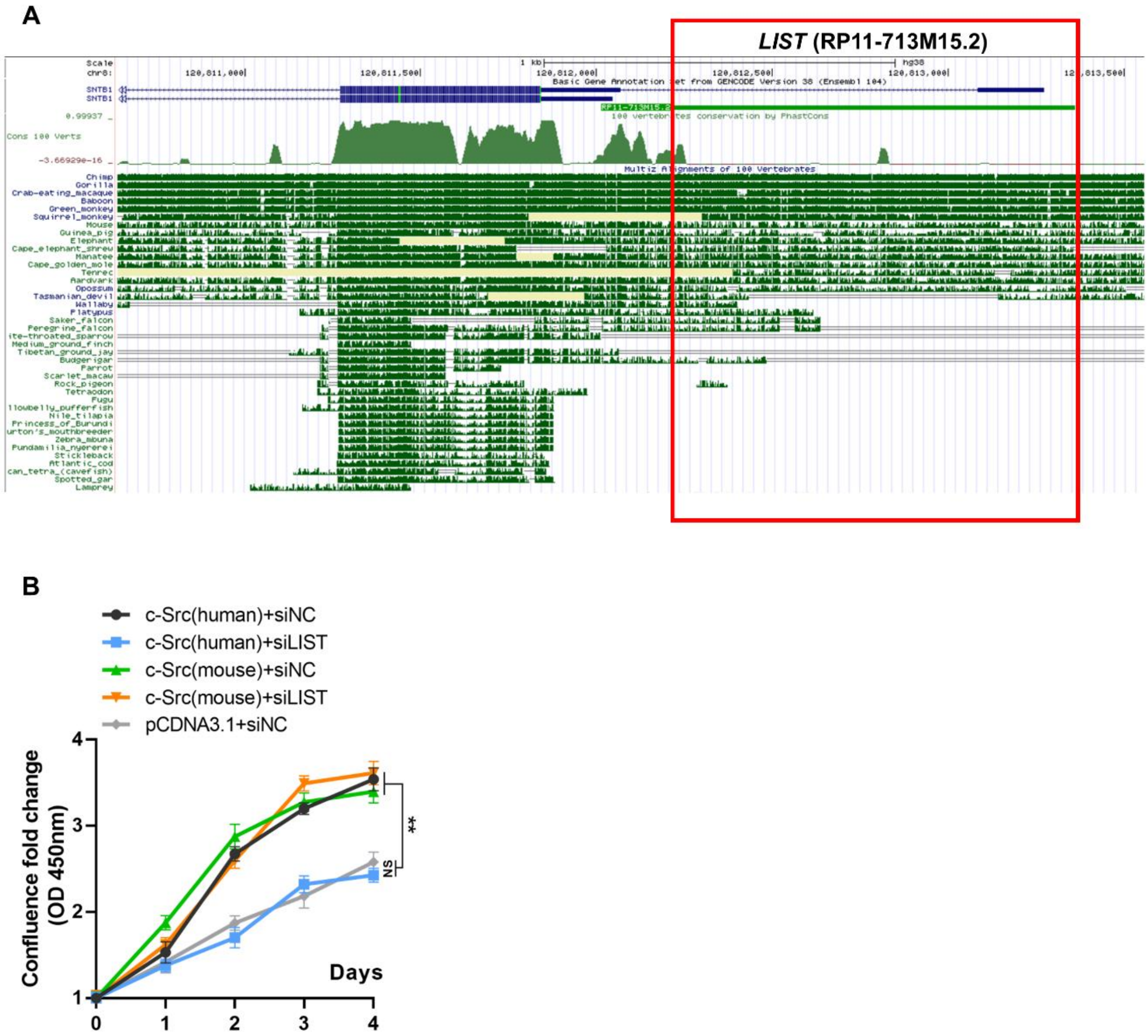

Fig. S20.

Cross-species conservation of *LIST* and comparison between mouse and human c-Src in terms of their dependency on *LIST*. (A). Genomic location of *LIST* on the GRCh38/hg38 reference and the cross-species conservation. Cons 100 Verts: Base-wide conservation by PhyloP across 100 vertebrates. Multiz alignments of some selected vertebrates are shown, including primates and other representative animals such as mouse, rat, and others. Image was generated with UCSC Genome Browser. (B). GFP-tagged human or mouse c-Src was re-expressed into c-Src$^{-/-}$cell line 5637-CEM. Cell proliferation curves were examined upon *LIST* knockdown. The error bars represent the ± SD of 3 biological replicates (**P-value < 0.01), Student's t-test.

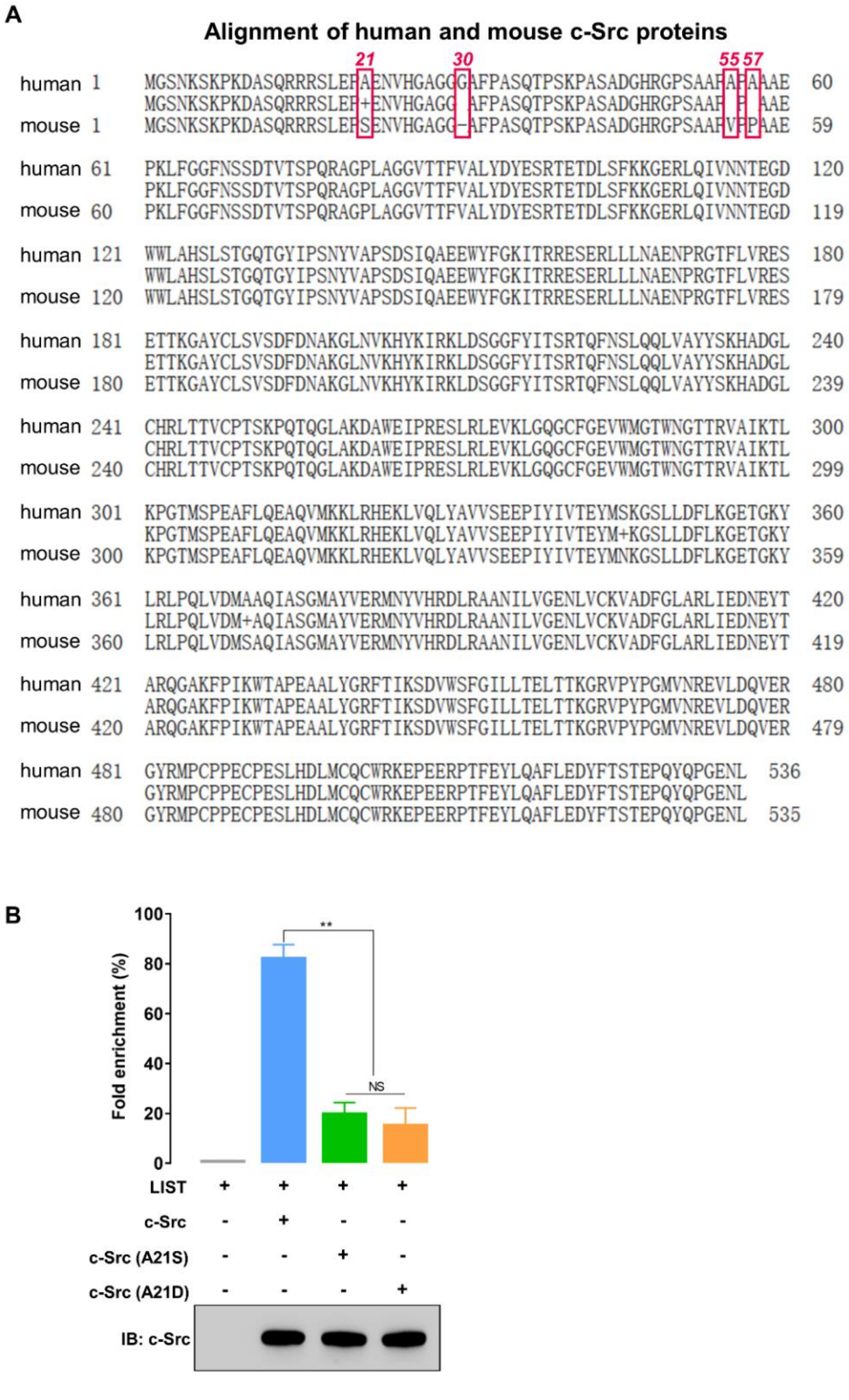

Fig. S21.

Comparison between mouse and human c-Src. (A). Alignment of the human and mouse c-Src proteins, generated by the NCBI protein blast tool. (B). In vitro RNA-protein binding assay. The purified wild-type or mutant human c-Src was incubated with *LIST* transcript. qRT-PCR of *LIST* was then performed with the material pulled down by anti-c-Src The error bars represent the ± SD of 3 biological replicates (**P-value < 0.01), Student's t-test.

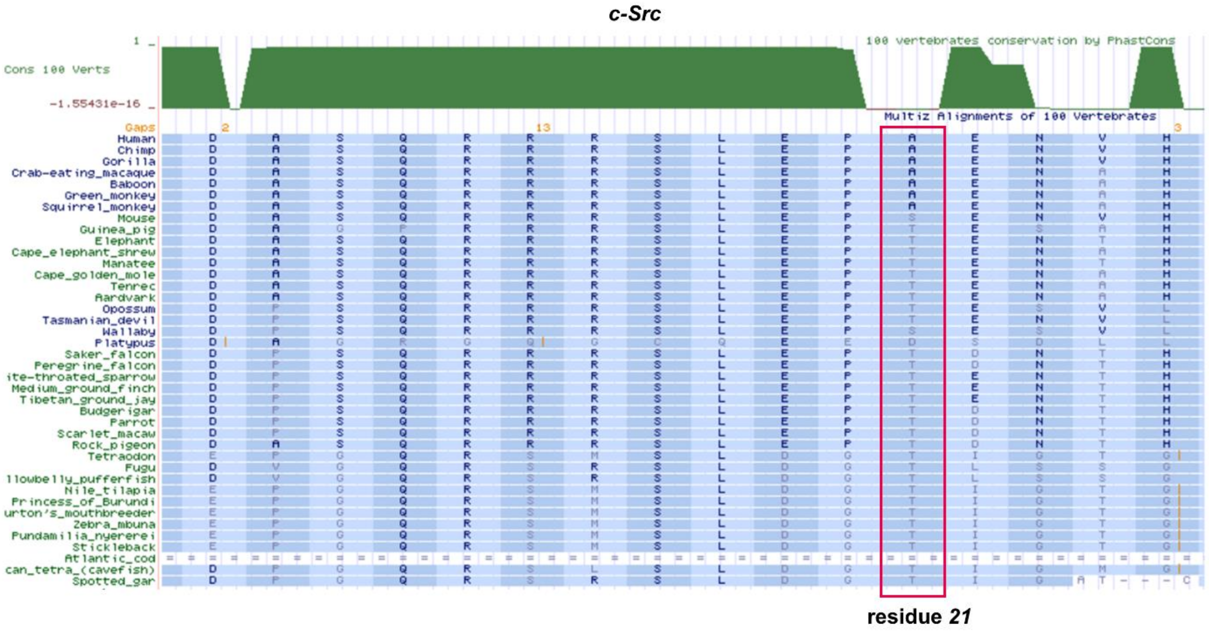

Fig. S22.

Conservation of c-Src sequence across species. Alignment of the c-Src amino acid sequences around the residues 21 across some representative species.

Table S1. Correlation between *LIST* expression and clinicopathological features in NSCLC patients (n=106)

Characteristics	Number	LIST		p-value
		High expression(n=53)	Low expression(n=53)	
**Gender (n, %)**				**0.051**
Female	48	29(60.4)	19(39.6)	
Male	58	24(41.4)	34(58.6)	
**Smoking**				**0.169**
Y	45	19(42.2)	26(57.8)	
N	61	34(55.7)	27(44.3)	
**TNM stage**				**0.006**
I-II	33	23(69.7)	10(30.3)	
III	73	30(41.1)	43(58.9)	
**Differentiation**				**0.052**
well moderate	54	32(59.3)	22(40.7)	
poor	52	21(40.4)	31(59.6)	
**Histologic type**				**0.506**
Adenocarcinoma	96	49(51)	47(49)	
Squamous carcinoma	10	4(40)	6(60)	

Table S2. Correlation between *LIST* expression and chemotherapeutic response in NSCLC patients (n=106)

Chemotherapy response	cases	*LIST* expression		χ^2	*P* Value
		Low	High		
platinum-resistant	51	13 (25.5%)	38 (74.5%)	23.619	P<0.001
platinum-sensitive	55	40 (72.7%)	15 (27.3%)		

Chi-square test was applied to assess the expression of *LIST* in platinum-resistant group and platinum-sensitive group

Table S3. Correlation between c-Src activity and chemotherapeutic response in NSCLC patients (n=106)

Chemotherapy response	cases	c-Src(active) expression		χ^2	*P* Value
		Low	High		
platinum-resistant	51	14 (27.4%)	37 (72.6%)	19.991	P<0.001
platinum-sensitive	55	39 (70.9%)	16 (29.1%)		

Chi-square test was applied to assess the expression of c-Src activity in platinum-resistant group and platinum-sensitive group

Protein	Signals (Ctrl)	Signals (DAS)	Ctrl/DAS
ATF2	452.50	211.34	2.14
BCL-XL	745.13	186.30	4.00
MEK1	1215.30	524.65	2.32
MKK4/SEK1 (Phospho-Thr261)	168.54	37.54	4.49
AKT1 (Phospho-Thr308)	253.14	93.29	2.71
AKT2 (Phospho-Ser474)	211.43	31.32	6.75
ATF2 (Phospho-Ser62/44)	163.23	78.33	2.08
BAD (Phospho-Ser136)	289.64	121.48	2.38
MEK1 (Phospho-Ser217)	198.73	41.34	4.81
MEK1 (Phospho-Ser221)	114.24	26.25	4.35
ASK1 (Phospho-Ser966)	235.30	98.12	2.40
Myc	834.20	411.54	2.03
p44/42 MAP Kinase	309.58	145.73	2.12
MAP3K7/TAK1 (Phospho-Thr184)	211.48	89.72	2.36
p44/42 MAP Kinase (Phospho-Tyr204)	178.29	57.37	3.11
SAPK/JNK (Phospho-Tyr185)	152.64	73.41	2.08
P38 MAPK (Phospho-Thr180/Tyr182)	283.67	71.86	3.95
NFkB-p100/p52	793.86	342.45	2.32
NFkB-p65	1749.54	799.47	2.19
IKK-beta	895.76	376.87	2.38
IKK-beta (Phospho-Tyr188)	185.50	78.45	2.36
IKK-beta (Phospho-Tyr199)	121.54	53.78	2.26
IKK-gamma (Phospho-Ser31)	89.54	31.12	2.88
NFkB-p105/p50 (Phospho-Ser337)	105.30	26.76	3.93
NFkB-p100/p52 (Phospho-Ser865)	94.34	39.27	2.40
PLCG1 (Phospho-Tyr783)	167.33	79.52	2.10
NFkB-p65 (Phospho-Ser311)	231.43	48.17	4.80
NFkB-p65 (Phospho-Ser529)	275.34	61.54	4.47
STAT1	972.47	411.23	2.36
Raf1 (Phospho-Ser259)	189.32	61.54	3.08
STAT1 (Phospho-Tyr701)	211.54	78.12	2.71
STAT2 (Phospho-Tyr690)	189.17	59.28	3.19
JAK1 (Phospho-Tyr1022)	217.84	103.42	2.11
JAK2 (Phospho-Tyr1007)	168.34	42.31	3.98
DVL	482.30	231.23	2.09
Catenin beta	734.29	312.65	2.35
GSK3 beta (Phospho-Ser9)	178.32	34.61	5.15
Catenin beta (Phospho-Thr41/Ser45)	213.54	54.47	3.92
FAK (Phospho-Ser910)	392.73	72.84	5.39
c-Abl (Phospho-Tyr412)	283.13	46.96	6.03
Elk1	735.68	297.89	2.47
FGFR1	1275.44	489.62	2.60
Caspase 3	794.36	317.48	2.50
HSP27	1107.47	452.54	2.45
Zap-70	592.93	211.30	2.81
Src (Phospho-Tyr419)	382.78	29.23	13.10

Table S4.

Several key signaling pathways were regulated by c-Src. The expression and activation of key signaling molecules were examined with or without c-Src inhibitor (DAS); the signal values of these proteins with DAS/Ctrl ratios greater than 2 were shown.

Table S5. Targeting sequence of shRNA

shRNA	Sense 5′-3′	Antisense 5′-3′
sh*LIST*-1	GCAATCCAGAAATGCCCACAT	ATGTGGGCATTTCTGGATTGC
sh*LIST*-2	GCAAGGTGTTGAGAGGAAATA	TATTTCCTCTCAACACCTTGC
shNC	ACGTGACACGTTCGGAGAAA	TTTCTCCGAACGTGTCACGT

Table S6. Targeting sequence of siRNA

oligos	Sense 5′-3′	Antisense 5′-3′
si*LIST*-1	UCCAGAAAUGCCCACAUAA	UUAUGUGGGCAUUUCUGGA
si*LIST*-2	GCAAGGUGUUGAGAGGAAA	UUUCCUCUCAACACCUUGC
siP65-1	GAUUGAGGAGAAACGUAAA	UUUACGUUUCUCCUCAAUC
siP65-2	GCAUCCAGACCAACAACAA	UUGUUGUUGGUCUGGAUGC
siHNF4A-AS1	GGUCAUAUUAUUGAGGUAA	UUACCUCAAUAAUAUGACC
siRP11-66N11.8	GGUUACUGAGGAAACCGAA	UUCGGUUUCCUCAGUAACC
siLINC00526	GGGCGGAGUCGCCGUUAAA	UUUAACGGCGACUCCGCCC
siPLGLA	CUCUGGAUGACUAUGUAAA	UUUACAUAGUCAUCCAGAG
siFAM99A	AGAGGUCAGAACCUCCCAA	UUGGGAGGUUCUGACCUCU
siFGF14-AS2	CCUCCUCCUCAGAGCCGAA	UUCGGCUCUGAGGAGGAGG
siABCC6P1	UCAGGUUCACUGUCCCCAA	UUGGGGACAGUGAACCUGA
siAC108488.4	GUAACUUGUCACUGUGGAA	UUCCACAGUGACAAGUUAC
siTP53TG1	CCUGACCCAGGAUCUAGAA	UUCUAGAUCCUGGGUCAGG
siSNHG9	CCCAUGUGCGGCGGUGAAA	UUUCACCGCCGCACAUGGG
siNC	ACGUGACACGUUCGGAGAA	UUCUCCGAACGUGUCACGU

Table S7. Targeting sequence of sgRNA

sgRNA	Sequence 5′-3′
sg1(c-Src)	GCCGCGGTGGCCGTCGGCCG
sg2(c-Src)	GGAGAGGCGTGCGGCCACAG
sg*LIST*-1	GGTGAGCGGCCGCTGGGTTG
sg*LIST*-2	GAAGTAAACCCAACCCAGCA
Scramble sgRNA-1	GAACGTTGGCACTACTTCAC
Scramble sgRNA-2	GCGCCTTAAGAGTACTCATC

Table S8. The sequence of regulatory element for “signal conductor”

Regulatory Element	Sequence 5′-3′
sg*LIST*-1	*GGTGAGCGGCCGCTGGGTTG*GTTTTAGAGCTAGAAATAGCAAGTTAAAATAAGGCTAGTCCGTTATCAACTTGAAAAAGTGGCACCGAGTCGGTGCCAGCGG**GGGCGCTCC**GAAGCTTACAAGAAGGACAGCACGAATAAAACCTGCGTAAATCCGCCCCATTTGTGTAAGGGTAGTGGGTCGAATTCCGCTCA**GGAGCGCCC**TTTTTT
sg*LIST*-2	*GAAGTAAACCCAACCCAGCA*GTTTTAGAGCTAGAAATAGCAAGTTAAAATAAGGCTAGTCCGTTATCAACTTGAAAAAGTGGCACCGAGTCGGTGCGGTTGG**GGGCGCTCC**GAAGCTTACAAGAAGGACAGCACGAATAAAACCTGCGTAAATCCGCCCCATTTGTGTAAGGGTAGTGGGTCGAATTCCGCTCA**GGAGCGCCC**TTTTTT

Note: The cDNA sequences of the sg*LIST* guide region (italic), the sgRNA handle (underlined), the P65 aptamer core (shadowed), and the aptamer stem (in bold) are indicated.

Table S9. Primers used in this study for qRT-PCR analysis

Gene	forward primer (5‘-3’)	reverse primer (5′-3′)
LIST	GTCCTCCTGAGCCGACTTCC	TCGGGCTACGTTGACTCTTCAT
NEAT1	TTCACCTGCTCTGGCTCTTG	GCCAGGCACCGTGTTATACT
P65	TCCACCTCGACGCATTGCTG	GGTGCTCAGGGATGACGTAAAGG
HOTTIP	CCTAAAGCCACGCTTCTTTG	TGCAGGCTGGAGATCCTACT
U6	GCTTCGGCAGCACATATACT	CGCTTCACGAATTTGCGTGT
H19	ATCGGTGCCTCAGCGTTCGG	TCCTCGCCGTCACACCG
HNF4A-AS1	ACCAAGAGGCTCTGCTAGGCT	GCAACAGAGGAGGCCTGTCTT
RP11-66N11.8	AATCCTCCTGGGCAGGCTCAGA	TGCTGTCCCCGTCCTTGTGA
LINC00526	GAGGAATCTGACTGCACGGT	TTGGGGAGAAAGCGGCGGCTA
PLGLA	CAGTGTCACTAAGAAGCAGCTG	CATTCCCAGTCTTGCACTCTG
FAM99A	CTGTCCTCCGCTGGAACCCT	ACAGTGACCTGACCCACACTGT
FGF14-AS2	ACACTCGGAACCCAGCTTCAC	TGAGAGGCGGGGAGTTGGTGT
ABCC6P1	TGGATGAGGAACCGCAGTGCAG	AGGTAGAATGGAACACCTGCC
AC108488.4	ACTACCTGAAGAGGGACCACG	CCTCCAAGAGCCAAACAGAATG
TP53TG1	CAGGTCTGGCTTACCACACGCT	TCAGACCTGCCAGCTCTCAGAG
SNHG9	GGCTATAAACGTCCGCGCCTCC	TTCACCGCCGCACATGGGGCAG
Spike in	TAAACAGGATACCCGTCATCTGAAAGT	ATCGCCGTCGCTGATGGAGT
GAPDH	GGTCACCAGGGCTGCTTTTA	TTCCCGTTCTCAGCCTTGAC
LIST (-1500/-1000)	ATCCCAGTCCCTACACTCTACCAG	CCAGCCCGCAGTCTTTCCAG
LIST (-1000/-500)	CTCCTGCTTCAGCACCTTCAC	ACCAATGGCTCGTTCTGCAGG
LIST (-500/0)	GTTGGCAGCTGCACTCAGGCTGGT	TCACCGCCAGCGTGCCTCCT
